# Sex-dependent epigenetic disruption of YY1 binding by prenatal BPA exposure downregulates *Matr3* and alters *Agap1* splicing in the offspring hippocampus

**DOI:** 10.1186/s13293-025-00744-1

**Published:** 2025-08-11

**Authors:** Pattanachat Lertpeerapan, Songphon Kanlayaprasit, Surangrat Thongkorn, Kasidit Kasitipradit, Pawinee Panjabud, Kwanjira Songsritaya, Thanawin Jantheang, Masanobu Morita, Takaaki Akaike, Valerie W. Hu, Depicha Jindatip, Thanit Saeliw, Tewarit Sarachana

**Affiliations:** 1 The Ph.D. program in Clinical Biochemistry and Molecular Medicine, Department of Clinical Chemistry, Faculty of Allied Health Sciences, Chulalongkorn University, Bangkok, 10330 Thailand; 2 Chulalongkorn Autism Research and Innovation Center of Excellence (ChulaACE), Department of Clinical Chemistry, Faculty of Allied Health Sciences, Chulalongkorn University, Bangkok, 10330 Thailand; 3 Department of Biotechnology and Biomedicine (DTU Bioengineering), Technical University of Denmark, 2800 Kgs. Lyngby, Denmark; 4 The M.Sc. Program in Clinical Biochemistry and Molecular Medicine, Department of Clinical Chemistry, Faculty of Allied Health Sciences, Chulalongkorn University, Bangkok, 10330 Thailand; 5 Department of Environmental Medicine and Molecular Toxicology, Tohoku University Graduate School of Medicine, Miyagi, 980-8575 Japan; 6 Department of Biochemistry and Molecular Medicine, School of Medicine and Health Sciences, The George Washington University, Washington, DC 20037 USA; 7 Department of Anatomy, Faculty of Medicine, Chulalongkorn University, Bangkok, 10330 Thailand

**Keywords:** Autism spectrum disorder, Bisphenol A, Alternative splicing, *Agap1*, RNA-binding protein, *Matr3*, Transcription factor YY1, Sex difference, Interactome, Transcriptome

## Abstract

**Background:**

Autism spectrum disorder (ASD) is a neurodevelopmental condition characterized by impairments in communication, social interaction, and behavior. Its etiology involves a combination of genetic and environmental factors, and it is more prevalent in males. Bisphenol A (BPA), an endocrine-disrupting chemical commonly found in plastics, has been linked to an increased risk of ASD. However, the molecular mechanisms by which BPA affects gene regulation remain poorly understood.

**Methods:**

In this study, hippocampal tissues were collected from rat offspring prenatally exposed to BPA at a dose of 5,000 µg/kg of maternal body weight, equivalent to the NOAEL. RNA sequencing was performed to identify genes exhibiting differential alternative splicing. ASD-related alternatively spliced genes were selected for validation using high-resolution melting (HRM) analysis. Ingenuity Pathway Analysis (IPA) was used to predict associated biological functions, diseases, and molecular interaction networks. Additionally, BPA-responsive transcription factors, RNA-binding proteins, splicing regulators, and differentially expressed genes were analyzed to identify upstream regulatory mechanisms. Gene expression was validated by qRT-PCR, and transcription factor binding was confirmed via chromatin immunoprecipitation-qPCR (ChIP-qPCR).

**Results:**

RNA-seq analysis revealed that prenatal BPA exposure altered the alternative splicing of ASD-related genes in the hippocampus, including *Agap1*, *Ap2b1*, and *Kifap3*. Gene ontology and pathway analyses indicated that differentially spliced genes were involved in mRNA splicing processes in males and neuronal functions in females, patterns that align with ASD-related phenotypes. ChIP-qPCR revealed sex-specific differences in YY1 transcription factor binding at the *Matr3* promoter, with males showing reduced binding, leading to downregulation of *Matr3* expression. Notably, *Agap1*, a target of MATR3, exhibited increased alternative splicing specifically in the hippocampus of male offspring.

**Conclusions:**

This study provides the first evidence that prenatal BPA exposure disrupts the alternative splicing and transcriptional regulation of ASD-related genes in offspring’s hippocampus in a sex-specific manner. These disruptions, particularly the YY1-mediated regulation of *Matr3* and downstream effects on *Agap1* splicing in males, offer new insights into the molecular mechanisms by which BPA may contribute to ASD pathogenesis.

**Supplementary Information:**

The online version contains supplementary material available at 10.1186/s13293-025-00744-1.

## Highlights

Prenatal BPA exposure disrupts the alternative splicing of the ASD-related genes, including *Agap1*, in a sex-specific manner.

The gene interactome network analysis revealed that BPA-responsive genes exhibit differential alternative splicing patterns, which are involved in neurological function and mRNA modification in a sex-dependent manner, with differential alternative spliced genes of males involved in RNA-binding protein functions.

Prenatal BPA exposure decreased the binding of YY1 to the promoter of *Matr3*, which is the alternative splicing regulator of *Agap1*, leading to downregulation specifically in male offspring’s hippocampi.

**Supplementary Information:**

The online version contains supplementary material available at 10.1186/s13293-025-00744-1.

## Abstract (plain language summary)

Autism spectrum disorder (ASD) is a neurodevelopmental disorder condition, caused by genetic and environmental factors, including bisphenol A (BPA), a chemical found in daily life. Previous studies have linked prenatal BPA exposure to an increased risk of ASD. However, the underlying mechanisms of how BPA could contribute to ASD pathogenesis remains unclear. In this study, we investigated whether prenatal BPA exposure disrupts the regulatory mechanisms of alternative splicing in genes associated with ASD. This study is the first to demonstrate that prenatal BPA exposure dysregulated the splicing of genes related to ASD, including *Agap1*, *Ap2b1*, and *Kifap3* in the hippocampus of offspring. Moreover, the differentially alternative spliced genes were associated with neurological functions and disorders, including cell trafficking, neuron development, and cognitive disorders. Furthermore, we found that the transcription factor YY1 bound to the promoter of the *Matr3* gene, which encodes an RNA-splicing regulator of *Agap1* mRNA, in a sex-dependent manner in response to prenatal BPA exposure. In male offspring, the recruitment of YY1 to the *Matr3* promoter was reduced, resulting in decreased *Matr3* expression, while in females, it remained unchanged. These findings indicate that prenatal BPA exposure disrupts *Agap1* splicing through YY1 and MATR3 in males, suggesting a potential mechanism that might contribute to the male bias of ASD, which warrants further investigation.

**Supplementary Information:**

The online version contains supplementary material available at 10.1186/s13293-025-00744-1.

## Introduction

Autism spectrum disorder (ASD) is a neurodevelopmental disorder characterized by social interaction and communication as well as restricted behavior [1]. The prevalence of ASD recently reported by the Centers for Disease Control and Prevention (CDC) in 2016, was about 1 in 54 in the USA [2] and raised to 1 in 31 in 2022 [3], and it occurred more frequently in boys than in girls [4]. Several studies reported that genetics plays an important role in causing ASD, but the mechanism of those changes in genes remains unknown. Previous research has shown that ASD candidate genes, such as *RORA*, may be involved in a feedback mechanism influenced by sex hormones that could contribute to the sex bias seen in ASD individuals [5, 6]. The finding also suggested that RORA plays a critical role in regulating genes associated with ASD, contributing to the disorder’s pathology toward male bias [7]. Several studies have revealed that modifications and alterations of genes associated with ASD have been identified [8, 9], including methylation [10–15], microRNA and non-coding RNA [16–22], proteins [23, 24], and alternative splicing [25–27]. Environmental factors, including chemical agents (heavy metals, pesticides, etc.), drugs (valproic acid, thalidomide, misoprostol, etc.), and maternal factors (advance maternal age, stress, infections, etc.), have also been associated with ASD [28]. Many compounds that disrupt the regulation of hormonal behavior, such as bisphenol A, can increase the risk of ASD [29].

Bisphenol A (BPA) is a synthetic xenoestrogen, one of the endocrine disruptors [30]. It is used as a substrate for hardening epoxy resin and polycarbonate, resulting in the production of food containers, baby bottles, food-canning linings, compact discs, and other products [31]. Due to BPA’s solubility of 120–300 mg/l, it is slightly to moderately toxic to fish and other water invertebrate life [32]. Additionally, BPA is also found in children with ASD more than in typically developed individuals [33]. In low-dose oral BPA studies in humans, it was revealed that BPA is rapidly absorbed in the gastrointestinal tract and conjugated with glucuronic acid in the liver. Then, conjugated BPA is cleared out of the body and leaves a small amount of free BPA in the body, which can alter the regulation of hormonal activities [34–37]. Free BPA in blood can circulate and cross the placenta [38, 39], potentially altering the development of the fetal brain [40–42].

During critical periods, including the stage of brain development, minor disruptions can lead to the dysregulation of genes that control human behavior [43]. BPA can interact with estrogen receptors and alter gene regulation through its pathway [35], leading to neurological disorders [44]. Previous research has shown that mice exposed to BPA exhibit dysregulation of the migration process in neuronal cells, resulting in abnormal neuron positioning and anomalous connectivity in the thalamus and cortex [38]. It has been observed that BPA can reduce the number of Purkinje cells and also alter the synapse generation of cerebellar neurons [45]. Previous studies on BPA exposure revealed that prenatal BPA exposure significantly disrupted the expression of genes in the prefrontal cortex, resulting in impaired cortical development, and leading to altered behaviors associated with ASD, including a deficit in social novelty [46, 47]. BPA exposure can also induce changes in the transcriptomic profile of the brain cells, including primary cortical neuronal cells and primary hippocampal neural stem cells, in a sex-specific manner [46, 48]. Transcriptional regulation also plays a crucial role in controlling the expression of genes and producing mRNA transcripts [49]. Alternative splicing occurs during gene expression, where the pre-mRNA (precursor to mRNA) can be spliced in various ways, resulting in different combinations of exons being included in the final mRNA transcript. These processes are essential for complex cellular functions and development [50]. The regulation of alternative splicing involves various interacting components, such as cis-acting elements, trans-acting factors, chromatin structure, RNA structure, and transcription processes [51]. Splicing regulatory elements (SREs) located in gene regions regulate alternative splicing by interacting with trans-acting factors. RNA-binding proteins (RBPs) play a crucial role in forming ribonucleoprotein complexes that determine RNA fates. Defects in RBP functions can lead to neurodegenerative disorders [52] and neurodevelopmental disorders [53]. Kim et al. (2021) found that BPA exposure dysregulated genes involved in alternative splicing in the retinoblastoma Y79 cell model [54]. Moreover, RNA sequencing analysis of testis cells of rats exposed to low-dose BPA showed differentially expressed genes involved in the inhibition of the spliceosome [55]. However, there is still a limited understanding of the effects of BPA on alternative splicing regulation in cells, especially in brain cells. Our previous study revealed that prenatal BPA exposure dysregulated ASD candidate genes in the hippocampus [56]. Moreover, it also altered ASD-related TFs and target gene expression in a sex-dependent manner, affecting synaptogenesis differently in male and female offspring [57]. However, it remains unclear how BPA exposure contributes to ASD, particularly regarding the exact mechanisms involving alternative splicing, RNA-binding proteins (RBPs), or transcription factors.

**Fig. 1:**
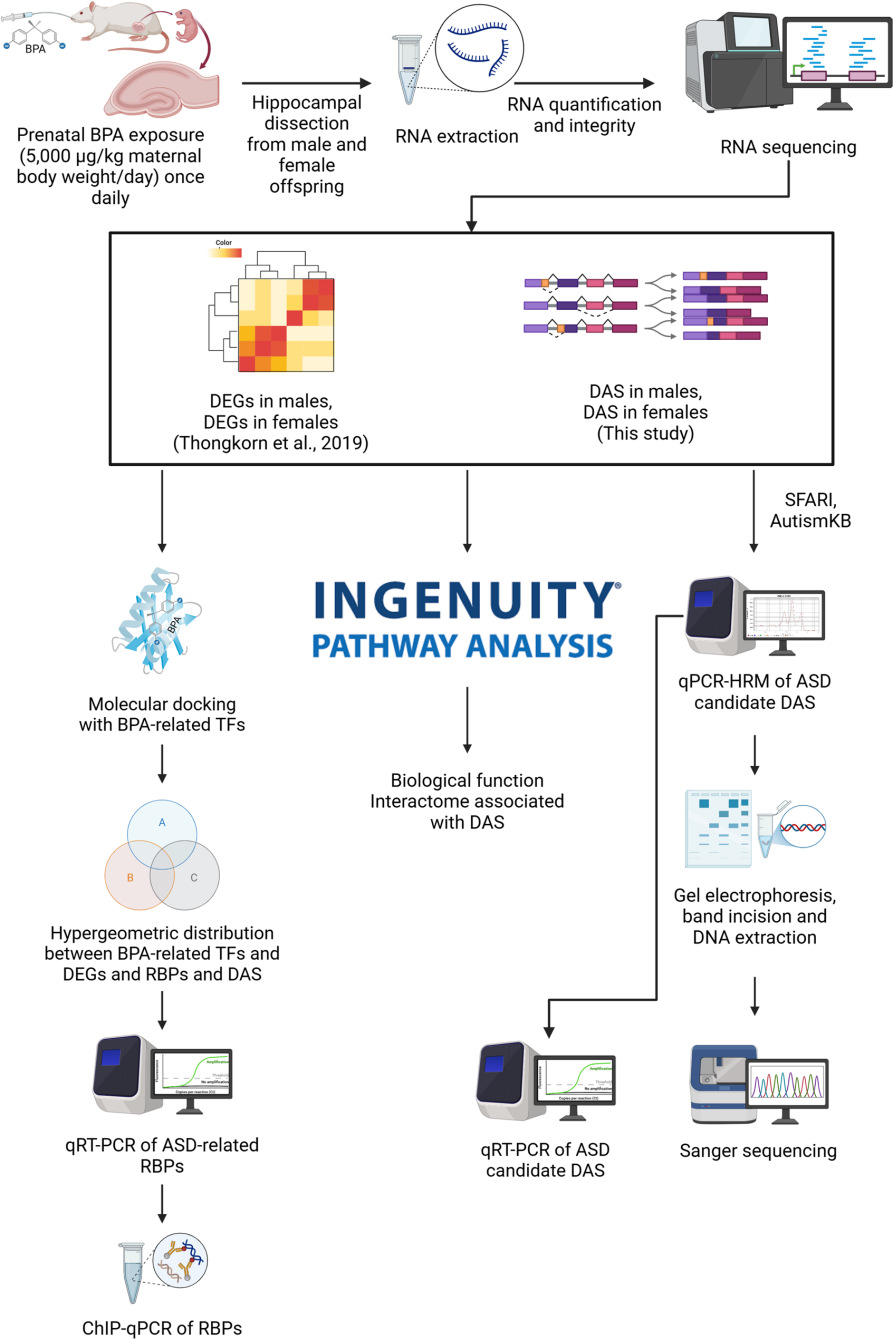
Schematic diagram of the experimental design. This figure was created in https://BioRender.com

In this study, we therefore investigated the effects of prenatal BPA exposure on alternative splicing and potential mechanisms in the hippocampus of neonatal rat offspring, as shown in Fig. 1.

## Methods

### Animal experiments

All procedures were conducted in full compliance with standard guidelines and regulations. The protocol was approved by the Chulalongkorn University Animal Care and Use Committee (Protocol no. 1673007, 1773011, 2073011, and 2273007). Eight-week-old male and female rats were purchased from the National Laboratory Animal Center (NLAC), Thailand, and housed at the Chulalongkorn University Laboratory Animal Center (CULAC), Thailand, under controlled conditions of temperature (21 ± 1 °C), humidity (30–70%), and a 12-hour light/dark cycle. Female rats (*n* = 6) were divided into 2 groups: the control group (*n* = 3) and the BPA treatment group (*n* = 3). We measured rat weight every day from gestational day 1 and calculated the BPA and vehicle amounts based on that weight. To prepare the BPA stock solution, BPA (Sigma-Aldrich, USA) was dissolved in ethanol (Merck Millipore, USA) at a concentration of 250 mg/ml. The stock solution was then diluted to 5,000 µg/kg of maternal body weight with corn oil and orally administered to the rats once daily. The vehicle control was prepared by mixing ethanol with corn oil in the same amount as the BPA treatment group. After mating, the treatment solution was administered intragastrically by oral gavage once a day from gestational day 1 until parturition. BPA-treated rats and control rats were raised separately to prevent chemical cross-contamination. Every piece of equipment for treatment or brain isolation was used in separate sets. After the maternal rat delivered rat offspring, neonate rat offspring were deeply anesthetized by intraperitoneal 100 mg/kg body weight sodium pentobarbital following hypothermia death on ice and confirmed death by decapitate. The brain was removed from the head and placed in prechilled 1X HBSS (Invitrogen, USA) containing 30 mM glucose (Sigma-Aldrich, USA), 2 mM HEPES (Cytiva, USA), and 26 mM NaHCO_3_ (Sigma-Aldrich, USA). The brains were then dissected, and the isolated hippocampus was examined under the microscope. Hippocampal tissues were then placed in a tube containing RNAlater (Thermo Fisher Scientific, USA) and stored at -80 °C for further procedures.

### RNA isolation and RNA-seq analysis

The RNA sequencing dataset, annotation, and DEGs used in this study were previously published in Thongkorn et al. 2019 [56]. In the present study, we reanalyzed this dataset to explore alternative splicing events. Briefly, male and female rat offspring were euthanized (for each sex, BPA *n* = 6 and control *n* = 6), and hippocampi were dissected, and then RNA isolation was performed. The total RNA from hippocampal tissues was isolated and purified using the mirVana miRNA Isolation Kit (Thermo Fisher Scientific, USA) as described in Thongkorn et al. (2019) [56]. The RNA was then analyzed using the Agilent Bioanalyzer (Agilent Technologies, USA) by BGI Group, China, for RNA integrity. RNA then performed RNA-seq using the Illumina HiSeq 4000 next-generation sequencing platform with 4G reads (Illumina, Inc., USA) as described in our previous study [56]. In this study, we re-analyzed the RNA-seq data published by Thongkorn et al. [56]. We also obtained the list of BPA-responsive genes in the hippocampi for hypergeometric distribution analysis. FASTQ files were then re-analyzed for the identification of alternative splicing events in the hippocampi of rat offspring prenatally exposed to BPA. Briefly, FASTQ files were aligned to the Rnor_6.0 reference genome using HISAT v0.1.6. The alternative splicing events were analyzed using the rMAT v3.0.9. The alternative splicing events with *p*-value < 0.05 and FDR < 0.05 were considered statistically significant or differential alternative splice genes (DAS) [58, 59].

### qRT-PCR HRM analysis and qRT-PCR

Alternatively spliced genes from RNA-seq analysis overlapped with ASD-associated genes from the ASD database (SFARI gene; https://gene.sfari.org/database/human-gene/, accessed on Sep 10th, 2024, AutismKB; http://db.cbi.pku.edu.cn/autismkb_v2, accessed on Jun 6th, 2019) [60, 61] Male and female rat offspring were euthanized (for each sex, BPA *n* = 12 and control *n* = 12), and hippocampi were dissected and then performed RNA isolation. First-strand cDNA was synthesized using a RevertAid First-Strand cDNA Synthesis Kit (Thermo Fisher Scientific, USA) following the manufacturer’s protocol. cDNA synthesis was done by incubating for 5 min at 25 °C, followed by 60 min at 42 °C, and ending with a 5-minute reaction at 70 °C. High-resolution melting analysis-quantitative polymerase chain reaction (HRM-qPCR) was performed using Precision Melt Supermix (Bio-Rad, USA) on the QuantStudio™ 5 Real-Time PCR System for Human Identification (Thermo Fisher Scientific, USA) according to the manufacturer’s protocol. Briefly, 1 µl of cDNA template was mixed with Precision Melt Supermix, forward primer, reverse primer, and nuclease-free water. The reaction was conducted under the following conditions: initial DNA denaturation at 95 °C for 2 min, followed by 45 cycles of denaturation at 95 °C for 10 s, annealing at 60 °C for 30 s, and extension at 72 °C for 30 s. All reactions were done in triplicate. High-resolution melting analysis was performed with heteroduplex formation at 95 °C for 30 s and 60 °C for 1 min, followed by melting curve analysis starting from 65 to 95 °C with 0.05 °C increments. The EVAGREEN system (Bio-Rad, USA) was used to analyze each gene-specific primer. Specific primers were designed using Primer3web (http://primer3.ut.ee/) [62] and confirmed with the UCSC In Silico PCR Genome Browser (https://genome.ucsc.edu/cgi-bin/hgPcr) [63] and sequences from Ensembl (https://asia.ensembl.org/index.html) [64]. The sequences of the forward and reverse primers for rat *Agap1*, *Ap2b1*, *Kifap3*, *Cyp20a1*, *Kif1b*, *Slc19a1*, and *Meis2* were listed in the **Additional file 1**. The peak height of each gene was considered 100% and used to normalize the data, with each peak calculated as a percentage. The values were compared between the BPA treatment and control groups using a paired t-test, with a *p*-value < 0.05 indicating statistical significance.

For qRT-PCR, amplification was performed using the Bio-Rad CFX Connect Real-Time System (Bio-Rad, USA) according to the manufacturer’s protocol. Briefly, 1 µl of cDNA template was mixed with iTaq Universal SYBR Green Supermix (Bio-Rad, USA), forward primer, reverse primer, and nuclease-free water. The reaction was conducted under the following conditions: initial DNA denaturation at 95 °C for 30 s, followed by 40 cycles of denaturation at 95 °C for 5 s, and annealing at 60 °C for 30 s. All reactions were done in triplicate. Melting analysis was performed starting from 65 to 95 °C with 0.2 °C increments. The SYBR Green system (Bio-Rad, USA) was used to analyze gene expression using gene-specific primers for each gene. The primers were also designed the same as previously described. The sequence of the forward and reverse qPCR primer for rat *Agap1*, *Ap2b1*, *Cyp20a1*, *Kif1b*, *Kifap3*, *Slc19a1*, *Meis2*,* Tnrc6a*, *Ybx2*, *Ythdf3*, *Elavl4*, *Tial1*, *Hnrnpc*, *Matr3*, *Sfpq*, *Enox1*, *Rbm10* and *Rn18s* are listed in Additional File 1. 18 S ribosomal RNA (*Rn18s*) was used as a reference gene for all gene expression analysis. Expression was quantified as the fold change using the ΔΔCt method (2^− ΔΔCt^).

### Validation of the sequences of alternative splicing variants

To validate the sequences of alternative splicing variants, gel electrophoresis of qPCR-HRM products was performed. DNA was then extracted from each band on the gel, purified, and sequenced using Sanger’s sequencing method by Macrogen (Macrogen, Inc., South Korea). The sequences were then aligned with the Rnor_6.0 reference genome in the UCSC Genome Browser (http://genome.ucsc.edu) [63].

### Prediction of biological functions and network analysis

Biological pathways, canonical pathways, and networks associated with the DAS genes were predicted using IPA software (Qiagen, Inc., Germany) [65]. The list of DAS genes was compared against genes in Ingenuity’s Knowledge Base Database. The *p*-value was calculated by the software using Fisher’s exact test, with a *p*-value < 0.05 considered statistically significant.

### Identification of RBPs and the DAS gene list associated with BPA

To identify BPA-responsive RBPs in the hippocampi prenatally exposed to BPA, we performed a hypergeometric distribution analysis between (1) BPA-related transcription factor gene lists Thongkorn et al., 2023 [57], (2) BPA-responsive genes that were published in the study of Thongkorn et al., 2019 [56], and (3) RBPs gene lists from the oRNAment database (https://rnabiology.ircm.qc.ca/oRNAment, accessed on Jul 8th, 2019) [66] and POSTAR2 database (http://lulab.life.tsinghua.edu.cn/postar, accessed on Jul 9th, 2019) [67] using Venny 2.1 software (https://bioinfogp.cnb.csic.es/tools/venny/, accessed on Jul 10th, 2019) [68] and the Keisan online Calculator package (http://keisan.casio.com/exec/system/1180573201, accessed on Jul 10th, 2019), respectively. We then obtained the target genes of those BPA-responsive RBPs from the oRNAment and POSTAR2 databases and overlapped with our DAS using hypergeometric distribution analysis. A *p*-value < 0.05 was considered a significant association.

### Chromatin immunoprecipitation and qRT-PCR (ChIP-qPCR)

ChIP was performed using a Chromatin Immunoprecipitation (ChIP) Assay Kit (Merck Millipore, USA) with minor modifications. Briefly, the hippocampi of each group were pooled in a total weight of ~ 180 mg, fixed with 37% formaldehyde, and incubated for 15 min at room temperature with agitation. Fixed tissues were then quenched with 2 ml of 10X glycine and incubated for 5 min at room temperature. The tissues were pelleted at 800 × g, 4 °C for 5 min, then resuspended with cold PBS. The tissues were homogenized with a Dounce homogenizer (loose pestle) on ice and then pelleted at 1,000 × g, 4 °C for 5 min. The supernatant was removed. The pelleted cells were resuspended in 200 µl of warmed SDS lysis buffer (Merck Millipore, USA) containing a protease inhibitor cocktail and incubated on ice for 10 min. The chromatin was sonicated to 200–1000 bp fragments using a Vibra-Cell VCX750 sonicator (Sonics & Materials, Inc., USA) with 20% amplitude for 15 cycles of 5 s on and 5 s off. The sonicated samples were reversed the cross-linked by adding 8 µl of 5 M NaCl and incubated at 65 °C for 4 h. The samples were centrifuged at 13,000 rpm at 4 °C for 10 min. Then, the samples were diluted 10-fold with ChIP Dilution Buffer (Merck Millipore, USA). A 20 µl aliquot of chromatin was kept at 4 °C overnight and used as input DNA. Each sample was incubated overnight with antibodies (3 µg/tube) to precipitate chromatin. The following primary antibodies were used: H3K27Me3 (Cell Signaling Technology, USA), ESR1 (Merck Millipore, USA), YY1 (Santa Cruz Biotechnology, USA), mouse IgG (Merck Millipore, USA), and rabbit IgG (Merck Millipore, USA). The chromatin-antibodies complexes were precipitated with protein A agarose/salmon sperm DNA (Merck Millipore, USA) and washed with the following buffer: low salt immune complex wash buffer (Merck Millipore, USA), high salt immune complex wash buffer (Merck Millipore, USA), LiCl immune complex wash buffer (Merck Millipore, USA), and TE buffer (Merck Millipore, USA). Samples were then eluted with elution buffer (1% SDS, 0.1 M NaHCO_3_). Lastly, DNA was extracted using the phenol-chloroform method. The ChIP enrichment analysis of DNA was performed using qRT-PCR. The primer sequences for 4 binding sites in *Matr3* and 2 binding sites of *Hnrnpc* were listed in Additional File 1.

### Statistical analysis

The statistical analysis was performed using the SPSS software package for Windows. The results were presented as mean ± S.E.M. The significance of differences between the means of the two groups in HRM analysis, as well as the expression levels of DAS, RBPs, and the enrichment of transcription factors in binding sites, was analyzed using a paired t-test. A *p*-value < 0.05 was considered a statistically significant difference.

## Results

### Prenatal BPA exposure dysregulated alternative splicing events in hippocampal tissues

**Fig. 2:**
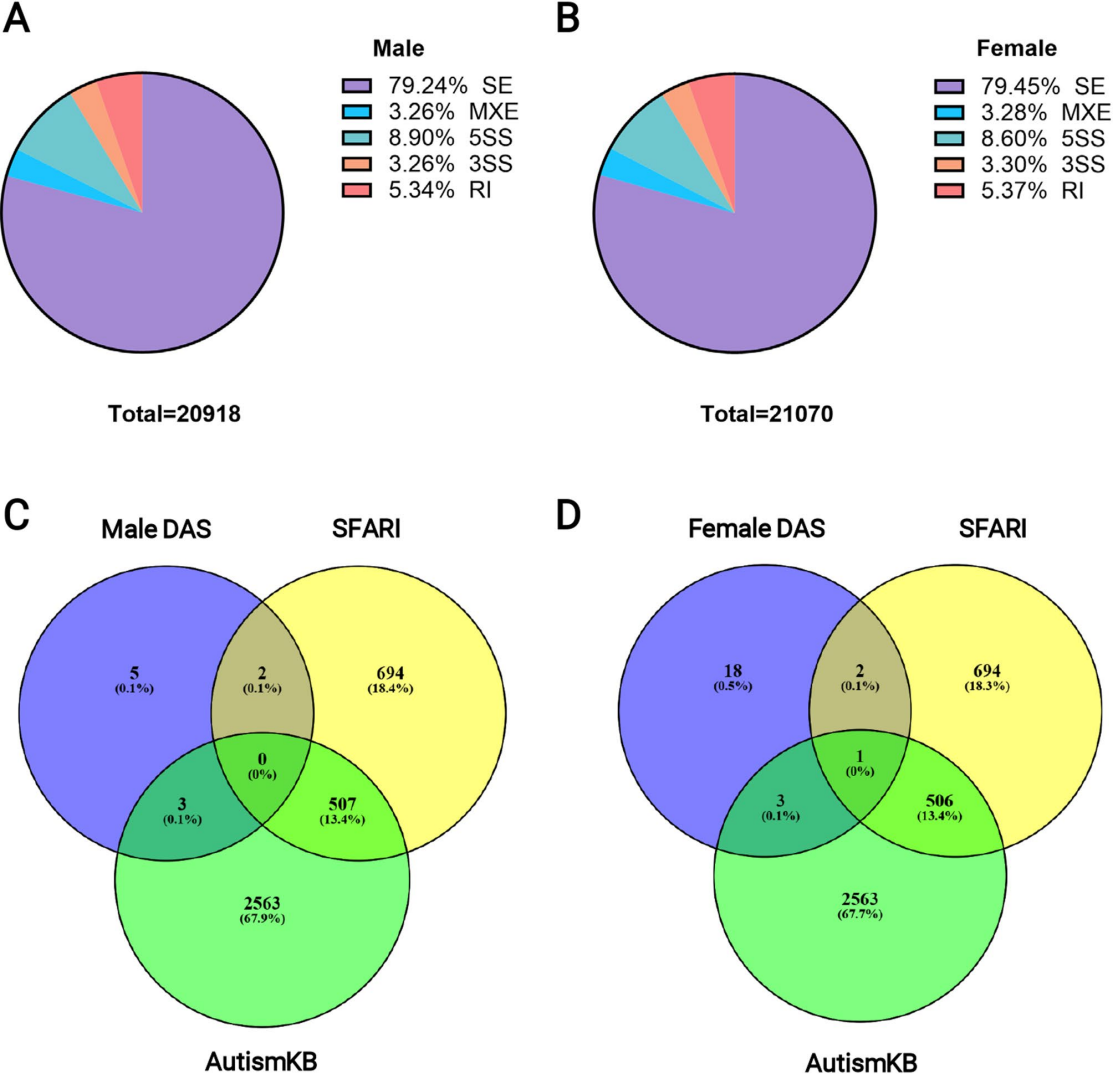
Alternative splicing events in the hippocampal tissues of rat offspring. (**A**) All detectable alternative spliced events in male rat offspring, (**B**) All detectable alternative spliced events in female rat offspring, (**C**) Venn diagram of DAS of males overlapped with SFARI and AutismKB, (**D**) Venn diagram of DAS of females overlapped with SFARI and AutismKB

Our previous study, conducted by Thongkorn et al. (2019), revealed that prenatal BPA exposure altered the expression of genes in the hippocampal tissues of the offspring. Briefly, we found that 2,078 genes and 3,522 genes were significantly differentially expressed in the male and female hippocampi, respectively [56]. In this study, we focus on the effects of prenatal BPA exposure on alternative splicing in the hippocampus. The results revealed 20,918 and 21,070 alternative splicing events in the hippocampal tissue of male and female rat offspring (Additional Files 2 and 3). Most alternative splice types were skipping exons (SE), followed by alternative 5’ (5SS), intron retention (RI), then alternative 3’ (3SS), and mutually exclusive exons (MXE), in equal proportions (Fig. 2A and B). We compared alternative splicing events between rat offspring prenatally exposed to BPA and vehicle control. The alternative splicing comparison of hippocampal tissue of rat offspring, including both males and females, revealed no significant differential alternative spliced genes (DAS). In the hippocampi of male rat offspring, there were 12 significant alternative splicing events corresponding to 11 DAS compared with the vehicle control group. In females, 25 significant alternative splicing events corresponding to 24 DAS were identified compared with their sex-matched vehicle control group (Table 1). Subsequently, we overlapped the DAS from each analysis with SFARI and AutismKB, which are the ASD databases. In males, the Venn diagram revealed two DAS genes that overlapped with SFARI: *Ubap2l* and *Hnrnpr*, and three DAS genes that overlapped with AutismKB were *Kif1b*, *Znf689*, and *Sh3kbp1*. In females, only one DAS gene overlapped with both SFARI and AutismKB: *Agap1*, two DAS genes overlapped with SFARI: *Meis2* and *Ubap2l*, and three DAS genes overlapped with AutismKB: *Cyp20a1*, *Kif1b*, and *Slc19a1* (Fig. 2C and D). This finding indicated that prenatal BPA exposure altered alternative splicing events in the hippocampi of rat offspring. Finally, we selected five genes: *Agap1*, *Ap2b1*, *Cyp20a1*, *Kif1b*, *Kifap3*, *Slc19a1*, and *Meis2* for alternative splicing validation.

**Table 1:** Differentially alternative spliced (DAS) genes in hippocampal tissues of male and female rat offspring

Male					
Splice type	Gene	Gene description	ΔPSI	*p*-value	FDR
Alternative 3’	*Lrp10*	LDL receptor related protein 10	-0.47	1.14E-06	1.28E-03
Mutually exclusive exons	*Kifap3*	Kinesin associated protein 3	-0.52	< 1.00E-15	< 1.00E-15
	*Kif1b*	Kinesin family member 1B	-0.90	< 1.00E-15	< 1.00E-15
	*Ubap2l*	Ubiquitin associated protein 2 like	-0.87	< 1.00E-15	< 1.00E-15
	*Sh3kbp1*	SH3 domain containing kinase binding protein 1	-0.46	1.30E-14	1.30E-14
	*Hnrnpr*	Heterogeneous nuclear ribonucleoprotein R	-0.27	3.64E-13	3.64E-13
	*Arl13b*	ADP ribosylation factor like GTPase 13B	-1.00	1.20E-11	1.20E-11
Intron retention	*C19orf25*	Chromosome 19 open reading frame 25	0.32	8.59E-09	5.86E-06
Skipping exon	BGI_novel_G003329		-0.30	4.40E-07	7.30E-03
	*Ap2b1*	Adaptor related protein complex 2 subunit beta 1	0.15	1.36E-06	1.13E-02
	*Znf689*	Zinc finger Protein 689	0.95	4.66E-06	2.58E-02
	*Arl13b*	ADP ribosylation factor like GTPase 13B	0.90	1.18E-05	4.89E-02

Female					
Splice type	Gene	Gene description	ΔPSI	*p*-value	FDR
Alternative 3’	*Meis2*	Meis homeobox 2	-0.50	5.71E-10	6.46E-07
	*Pik3cd*	Phosphatidylinositol-4,5-bisphosphate 3-kinase catalytic subunit delta	0.47	5.66E-05	3.20E-02
Alternative 5’	*Ankrd10*	Ankyrin repeat domain 10	-0.04	1.77E-08	1.23E-05
	*Slc15a4*	Solute carrier family 15 member 4	-0.48	1.25E-04	4.36E-02
Mutually exclusive exons	*Kif1b*	Kinesin family member 1B	0.91	< 1.00E-15	< 1.00E-15
	*Ubap2l*	Ubiquitin associated protein 2 like	0.95	< 1.00E-15	< 1.00E-15
	*Rhot1*	Ras homolog family member T1	0.33	1.65E-07	9.98E-05
	*Gyg1*	Glycogenin 1	-0.28	2.65E-06	1.20E-03
	*Lypd6b*	LY6/PLAUR domain containing 6B	-0.76	1.45E-05	5.28E-03
Intron retention	*Hnrnpdl*	Heterogeneous nuclear ribonucleoprotein D like	-0.12	1.48E-08	1.02E-05
	*Ankrd10*	Ankyrin repeat domain 10	-0.03	8.29E-08	2.86E-05
	*C19orf25*	Chromosome 19 open reading frame 25	-0.28	1.52E-06	3.50E-04
	*Slc19a1*	Solute carrier family 19 member 1	-0.55	3.97E-05	6.86E-03
	*Kctd17*	Potassium channel tetramerization domain containing 17	-0.11	9.42E-05	1.19E-02
	*Ogfr*	Opioid growth factor receptor	-0.17	1.03E-04	1.19E-02
Skipping exon	*Hnrnpc*	Heterogeneous nuclear ribonucleoprotein C	0.49	2.57E-10	4.30E-06
	*Ints10*	Integrator complex subunit 10	-0.21	2.26E-08	1.26E-04
	*Ubxn2b*	UBX domain protein 2B	-0.21	2.25E-08	1.26E-04
	*Agap1*	ArfGAP With GTPase domain, ankyrin repeat, and PH Domain 1	-0.25	1.30E-07	5.45E-04
	*Cyp20a1*	Cytochrome P450 family 20 subfamily A member 1	-0.46	2.05E-06	6.85E-03
	*Mtg2*	Mitochondrial ribosome associated GTPase 2	-0.16	6.19E-06	1.73E-02
	*Mtg2*	Mitochondrial ribosome associated GTPase 2	-0.20	9.28E-06	2.22E-02
	*Abt1*	Activator of basal transcription 1	-0.22	1.07E-05	2.24E-02
	*Maf1*	MAF1 homolog, a negative regulator of RNA polymerase III	-0.10	1.79E-05	3.34E-02
	*Loc100912071*		-0.81	2.75E-05	4.60E-02

The figures in this table were created in https://BioRender.com

### BPA altered alternative spliced events of *Agap1*, *Ap2b1*, and *Kifap3* in the hippocampal tissues of rat offspring

**Fig. 3:**
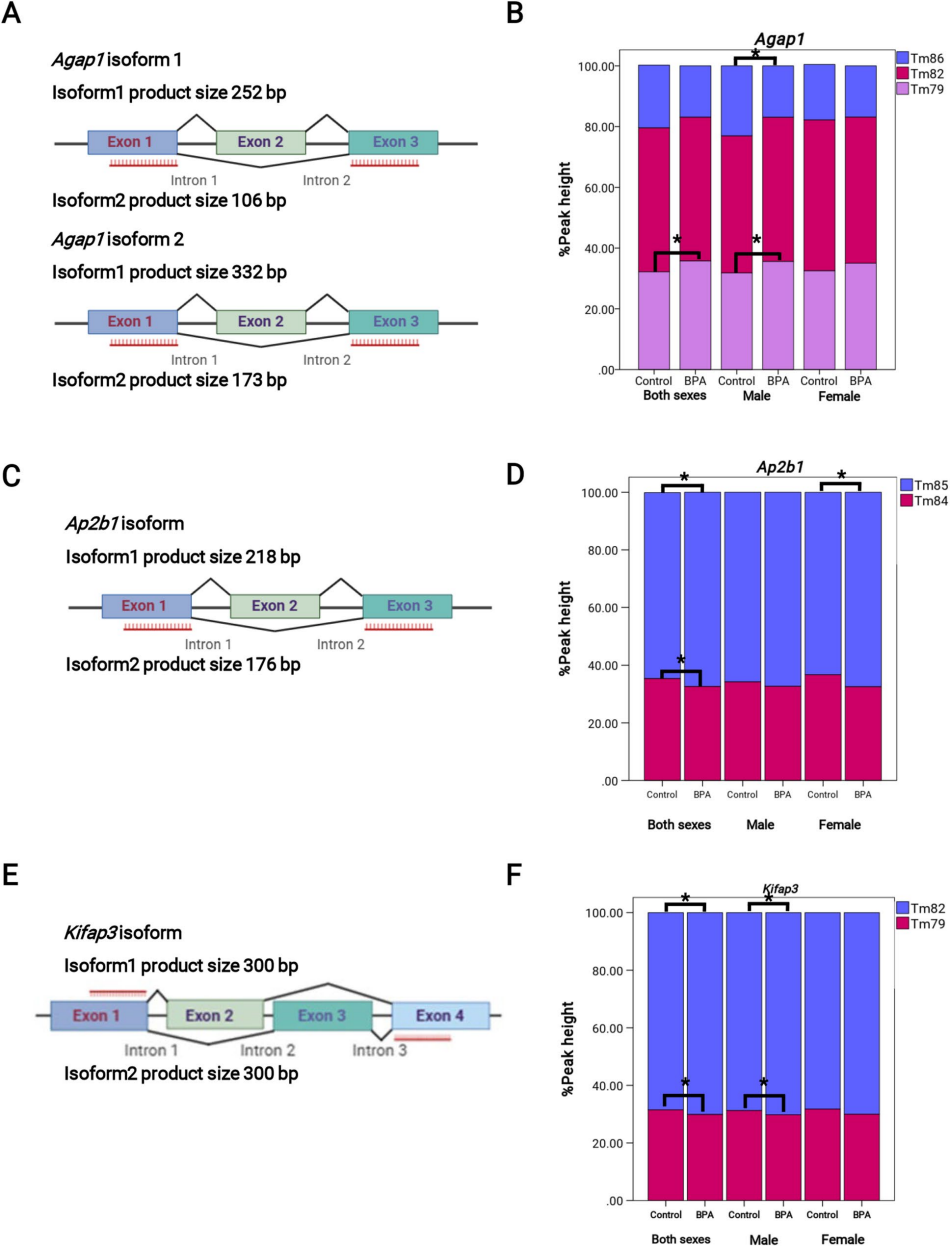
Differential alternative spliced genes detection of the hippocampal tissues of rat offspring. (**A**) Schematic figure of primer design for *Agap1* in quantification study created in https://BioRender.com, (**B**) High-resolution melting analysis of *Agap1* isoform using % peak height of each product (Both sexes Tm 79°C *p*-value = 0.047, Tm 82 °C *p*-value = 0.795, Tm 86°C *p*-value = 0.141, Male Tm 79°C *p*-value = 0.018, Tm 82 °C *p*-value = 0.192, Tm 86 °C *p*-value = 0.012, Female Tm 79°C *p*-value = 0.406, Tm 82 °C *p*-value = 0.638, Tm 86 °C *p*-value = 0.808), (**C**) Schematic figure of primer design for *Ap2b1* in quantification study created in https://BioRender.com, (**D**) High-resolution melting analysis of *Ap2b1* isoform using % peak height of each product (Both sexes Tm84°C *p*-value = 0.016, Tm 85 °C *p*-value = 0.010, Male Tm 84°C *p*-value = 0.143, Tm 85 °C *p*-value = 0.143, Female Tm 84°C *p*-value = 0.063, Tm 85 °C *p*-value = 0.038), (**E**) Schematic figure of primer design for *Kifap3* in quantification study created in https://BioRender.com, (**F**) High-resolution melting analysis of *Kifap3* isoform using % peak height of each product (Both sexes Tm 79°C *p*-value = 0.004, Tm 82 °C *p*-value = 0.004, Male Tm 79°C *p*-value = 0.046, Tm 82 °C *p*-value = 0.046, Female Tm 79°C *p*-value = 0.064, Tm 82 °C *p*-value = 0.064),* *p*-value < 0.05 paired t-test

We designed primers to detect alternative splicing using in-silico PCR from UCSC and found that our primers could bind to alternatively spliced sites, as shown in the schematic figure (Fig. 3A and C, and E). Consistent with prediction using in-silico PCR, qPCR-HRM analysis revealed three different *Agap1* PCR products with melting temperatures (Tm) of 79 °C, 82 °C, and 86 °C. We analyzed the skipping exon product (Tm = 79 °C) of both sexes combined and found that the skipping exon product of the BPA group was significantly increased compared to the control. We then analyzed the normalized percentage peak height of skipping exon and inclusion exon product (Tm = 86 °C) of males and females separately. We found a significantly increase in skipping exon product (Tm = 79 °C) corresponding to a significantly decrease in inclusion exon product (Tm = 86 °C) in the hippocampal tissues of male rat offspring only, but not in females (Fig. 3B). Moreover, the qPCR-HRM analysis of *Ap2b1* revealed two distinct melting temperatures at the skipping exon product (Tm = 84 °C) and the inclusion exon product (Tm = 85 °C). The analysis of the percentage peak height of the hippocampal tissues prenatally exposed to BPA of both sexes combined revealed a decrease in skipping exon product (Tm = 84 °C) corresponding to an increase in inclusion exon product (Tm = 85 °C). We also analyzed females separately and found a significant increase in inclusion exon product (Tm = 85 °C) in the BPA-treated group compared to the control, but not in males (Fig. 3D). Furthermore, we also performed the analysis of *Kifap3* and found skipping exon product (Tm = 79 °C) and inclusion exon product (Tm = 82 °C). The hippocampal tissues exposed to BPA of both sexes combined revealed a significant increase in inclusion exon product (Tm = 82 °C) corresponding to a significant decreased in skipping exon product (Tm = 79 °C). Interestingly, male hippocampal tissues exposed to BPA prenatally also showed a significant increase in inclusion product with a significant decrease in skipping exon product, but not in females (Fig. 3F). In contrast, the results of the comparison between BPA and control in both sexes combined, males and females, of the PCR product in *Cyp20a1* revealed no significant changes (Supplementary Fig. 1A). However, the HRM technique was unable to detect alternatively spliced forms of *Kif1b*, *Slc19a1*, and *Meis2* due to sequence similarities, intron retention, and splice site variations affecting primer efficiency. Moreover, we also confirmed the sizes of alternative *Agap1* products using gel electrophoresis. The gel electrophoresis of *Agap1* PCR products revealed that the PCR of *Agap1* contained four products. The product sizes were 106 bp, 173 bp, 252 bp, and 332 bp (Supplementary Fig. 2A). The BLAT analysis of the sequenced products against Rnor6.0 in the UCSC database revealed that the sequences from HRM matched *Agap1* variants in each product (Supplementary Fig. 2B). However, the 106 bp product was too short to be sequenced. Thus, the sequencing results indicated that the designed primers were specific to the *Agap1* gene and could detect alternative spliced variants of *Agap1*. The gel electrophoresis of *Ap2b1* revealed two amplicons, as expected from in-silico PCR (skipping: 176 bp and inclusion: 218 bp). The BLAT analysis of the sequenced products also revealed the HRM product matched with *Ap2b1* variants (Supplementary Figs. 3A and B). Moreover, the gel electrophoresis of *Kifap3* revealed only 1 amplicon (300 bp), and the BLAT analysis of the sequenced product revealed a it matched *Kifap3* in the UCSC database (Supplementary Figs. 4A and B). We also performed these analyses for *Cyp20a1* (Supplementary Figs. 1B and 1C). These results indicated that prenatal BPA exposure alters alternative splicing events of *Agap1* associated with ASD in the hippocampal tissues of rat offspring in a sex-dependent manner, particularly in males.

### BPA exposure did not dysregulate the expression of DAS genes

**Fig. 4:**
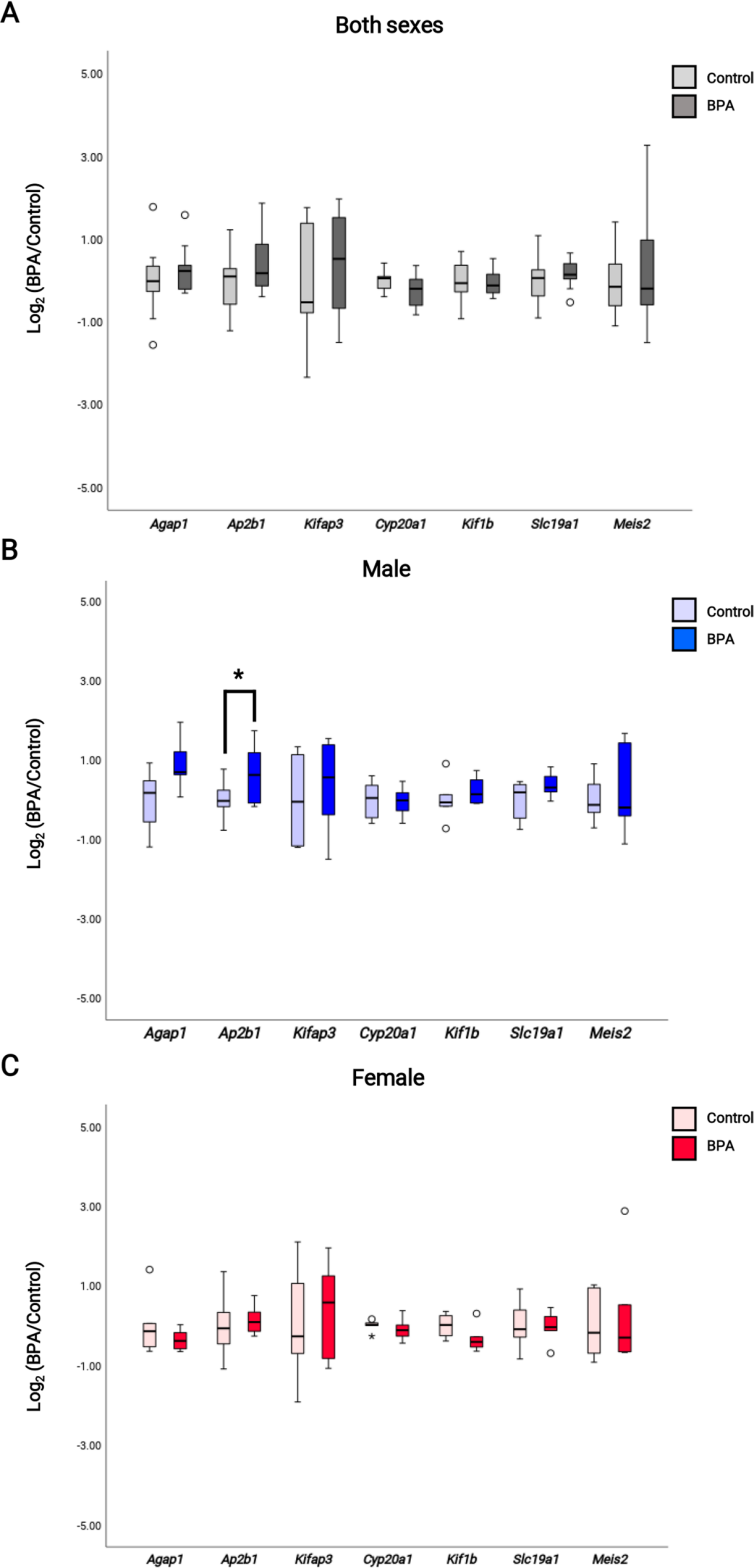
Boxplot of DAS expression analysis in the hippocampal tissues of rat offspring. (**A**) Quantitative RT-PCR analysis of the hippocampal tissues of both sexes rat offspring prenatally exposed to BPA or vehicle control was performed to assess the expression of *Agap1* (*p*-value = 0.454), *Ap2b1* (*p*-value = 0.083), *Cyp20a1* (*p*-value = 0.424), *Kif1b* (*p*-value = 0.776), *Kifap3* (*p*-value = 0.157), *Slc19a1* (*p*-value = 0.468), and *Meis2* (*p*-value = 0.572), (**B**) Quantitative RT-PCR analysis of the hippocampal tissues of male rat offspring prenatally exposed to BPA or vehicle control was performed to assess the expression of *Agap1* (*p*-value = 0.100), *Ap2b1* (*p*-value = 0.036), *Cyp20a1* (*p*-value = 0.731), *Kif1b* (*p*-value = 0.444), *Kifap3* (*p*-value = 0.218), *Slc19a1* (*p*-value = 0.145), and *Meis2* (*p*-value = 0.758), (**C**) Quantitative RT-PCR analysis of the hippocampal tissue of female rat offspring prenatally exposed to BPA or vehicle control was performed to assess the expression of *Agap1* (*p*-value = 0.394), *Ap2b1* (*p*-value = 0.687), *Cyp20a1* (*p*-value = 0.482), *Kif1b* (*p*-value = 0.206), *Kifap3* (*p*-value = 0.420), *Slc19a1* (*p*-value = 0.928), and *Meis2* (*p*-value = 0.653)

Gene expression analysis of the DAS genes by qRT-PCR analysis revealed no significant changes in *Agap1*, *Cyp20a1*, *Kif1b*, *Kifap3*, *Slc19a1*, and *Meis2* expression in both sexes, males and females (Fig. 4A, B, and C). In contrast, *Ap2b1* expression was significantly increased in male hippocampal tissues compared with the control group. These results indicated that prenatal BPA exposure caused an alteration in the alternative splicing of these genes, but not their expression, in the hippocampal tissues of rat offspring.

### DAS in the hippocampus were associated with biological functions and networks related to ASD

To determine the biological functions and networks of the DAS using Ingenuity Pathway Analysis (IPA) software. The software revealed that the biological functions of these DAS genes shown in Table 2 were mostly related to neurological function, hereditary disorders, and psychological disorders, which are highly relevant to ASD. For male offspring, we found that the function of those DAS genes was associated with “developmental disorder,” “hereditary disorder,” and “neurological diseases.” For females, DAS genes were mainly associated with “cancer” and “developmental disorder.” Moreover, we found that neurological disease categories of male rat offspring showed several diseases implicated with ASD, such as neurosensory-motor disorder and development disorders. In comparison, the DAS of female rat offspring showed mental retardation, which is associated with ASD (Table 3). These results suggested that prenatal BPA exposure disrupted the alternative splicing of genes associated with biological function and co-morbid disorders, which are implicated in ASD.

**Table 2:** Top diseases and disorders associated with differentially spliced genes in the hippocampal tissues of rat offspring

Male		
Categories	*p*-value	The number of genes
Developmental Disorder	1.88E-02–4.98E-04	2
Hereditary Disorder	1.88E-02–4.98E-04	2
Neurological Disease	3.98E-03–4.98E-04	2
Organismal Injury and Abnormalities	1.88E-02–4.98E-04	4
Renal and Urological Disease	1.88E-02–4.98E-04	2

Female		
Categories	*p*-value	The number of genes
Cancer	4.90E-02–1.05E-03	4
Cardiovascular Disease	1.05E-03–1.05E-03	1
Connective Tissue Disorders	7.30E-03–1.05E-03	3
Developmental Disorders	4.90E-02–1.05E-03	3
Gastrointestinal Disease	1.05E-03–1.05E-03	1

**Table 3:** Neurological diseases and functions of differentially spliced genes in hippocampal tissues of rat offspring

Male		
Diseases or functions annotation	*p*-value	Molecules
Joubert syndrome type 8	4.98E-04	*Arl13B*
Charcot-Marie-Tooth disease type 2A1	4.98E-04	*Kif1b*
Autosomal dominant neuroblastoma	9.95E-04	*Kif1b*
Spinal muscular atrophy proximal adult dominant	9.95E-04	*Kif1b*
Autosomal dominant pheochromocytoma	3.98E-03	*Kif1b*

Female		
Diseases or functions annotation	*p*-value	Molecules
Charcot-Marie-Tooth disease type 2A1	1.05E-03	*Kif1b*
Spinal muscular atrophy proximal adult dominant	2.09E-03	*Kif1b*
Myoclonic dystonia type 26	1.05E-03	*Kctd17*
Cleft palate, cardiac defects, and mental retardation	1.05E-03	*Meis2*
Neuroblastoma	8.53E-03	*Kif1b*,* Pik3cd*

**Fig. 5:**
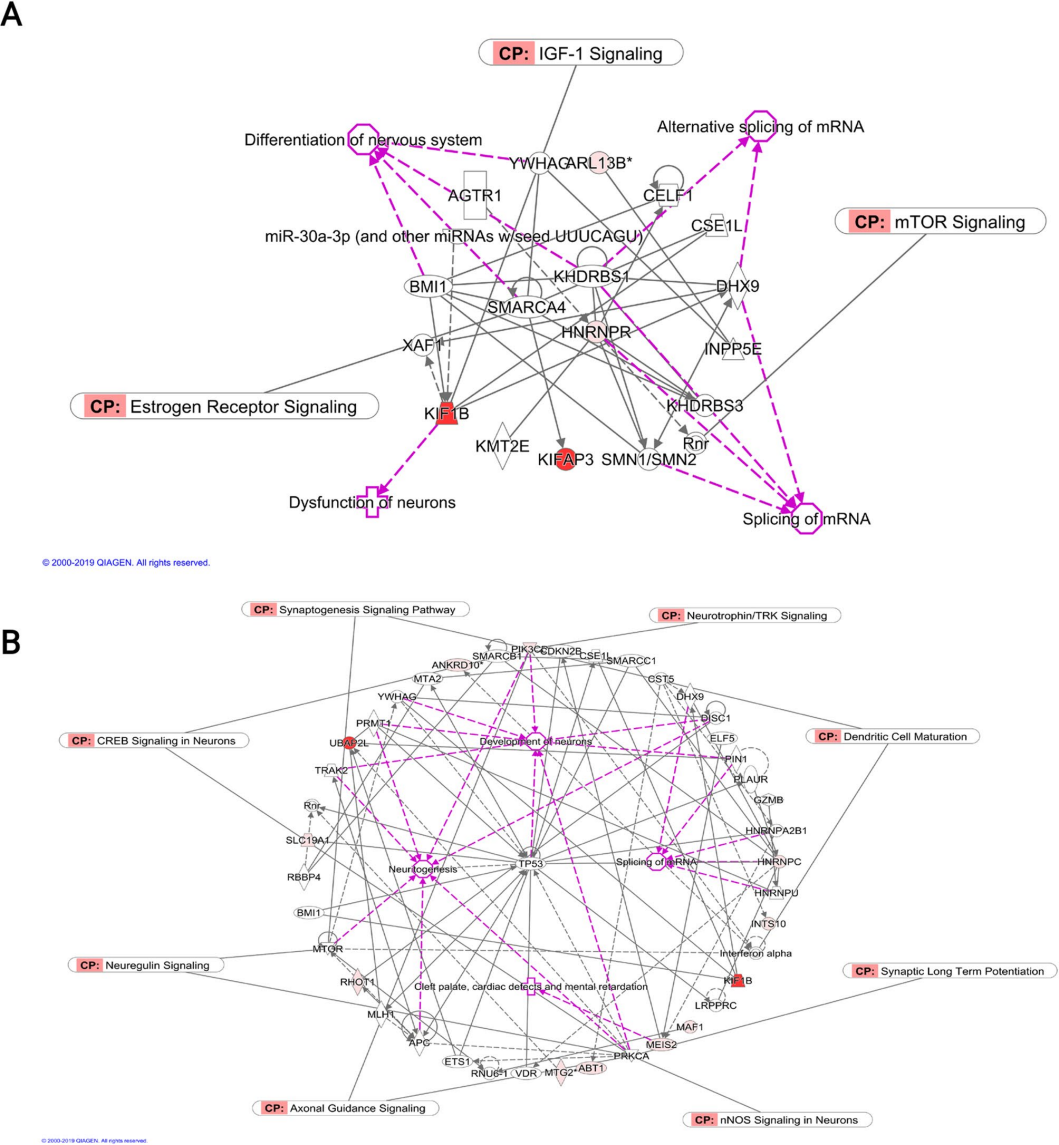
The biological network of alternatively spliced genes of hippocampal tissues. (**A**) male and (**B**) female, the differentially spliced genes (colored) were used to create the regulatory network. The DAS were predicted to be associated with neurological function (pink label) known to be associated with ASD, Fx; Function annotation, CP; canonical pathway (red represents to event count of alternative spliced genes)

The biological function analysis revealed networks and interactions of genes related to biological functions in neuritogenesis, dendritic growth/branching, migration of neurons, and synaptic transmission, which are associated with ASD. Moreover, the hippocampal tissues of male rat offspring revealed a network in which *Smarca4*, *Hnrnpr*, and *Khdrbs1* were hub genes; these genes are known for their roles in RNA transcription, transcription activation, and RBP functions, which are associated with alternative splicing processes. Interestingly, the networks revealed biological functions corresponding to alternative splicing of mRNA and estrogen receptor signaling, which is associated with the alteration of prenatal BPA exposure (Fig. 5A). In females, representative networks of DAS genes revealed the biological functions of neuritogenesis, neuronal development, and mental retardation. Moreover, those genes in those biological functions revealed essential signaling pathways in ASD individuals: CREB signaling in neurons, neuregulin signaling, axonal guidance signaling, synaptogenesis signaling pathway, GABA receptor signaling, synaptic long-term depression, and potentiation. One of the canonical pathways in the representative network corresponds to the estrogen receptor signaling pathway, which is known for BPA disruption. Those genes in function showed canonical pathways corresponding to synapse formation, dendritic maturation, and development (Fig. 5B). These results also suggested that prenatal BPA exposure affected DAS genes and their functions in a sex-dependent manner, as seen in the behaviors of individuals with ASD.

### DAS were the target of BPA-responsive RNA-binding proteins (RBPs)

**Fig. 6:**
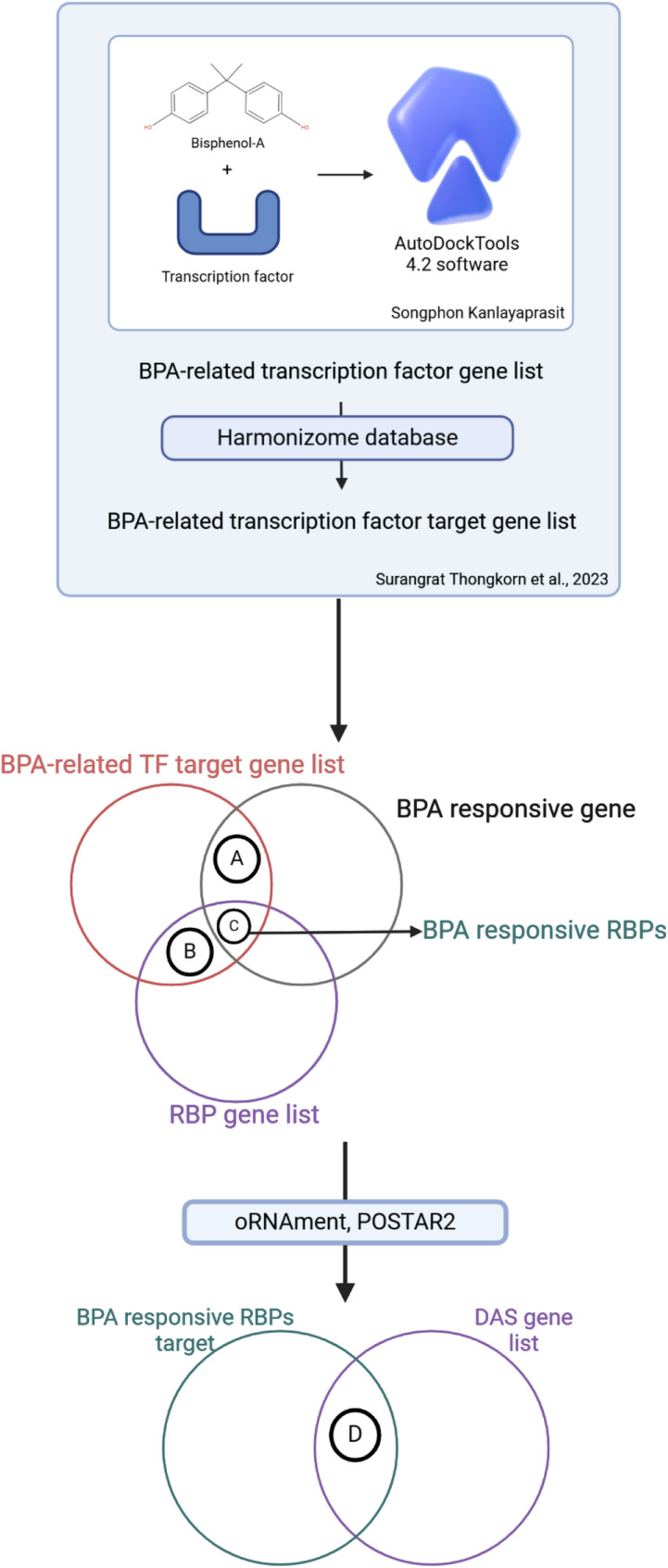
Schematic diagram illustrating the method used to identify BPA-responsive RBPs associated with DAS genes. The diagram shows the approach adapted from Thongkorn et al. (2023) [57] for identifying targets of ASD-related transcription factors and their overlaps with BPA-related DEGs (**A**) and RBPs (**B**). It also depicts the BPA-responsive RBPs that are targets of ASD-related transcription factors (**C**) and our DAS gene list (**D**). This figure was created in https://BioRender.com

**Fig. 7:**
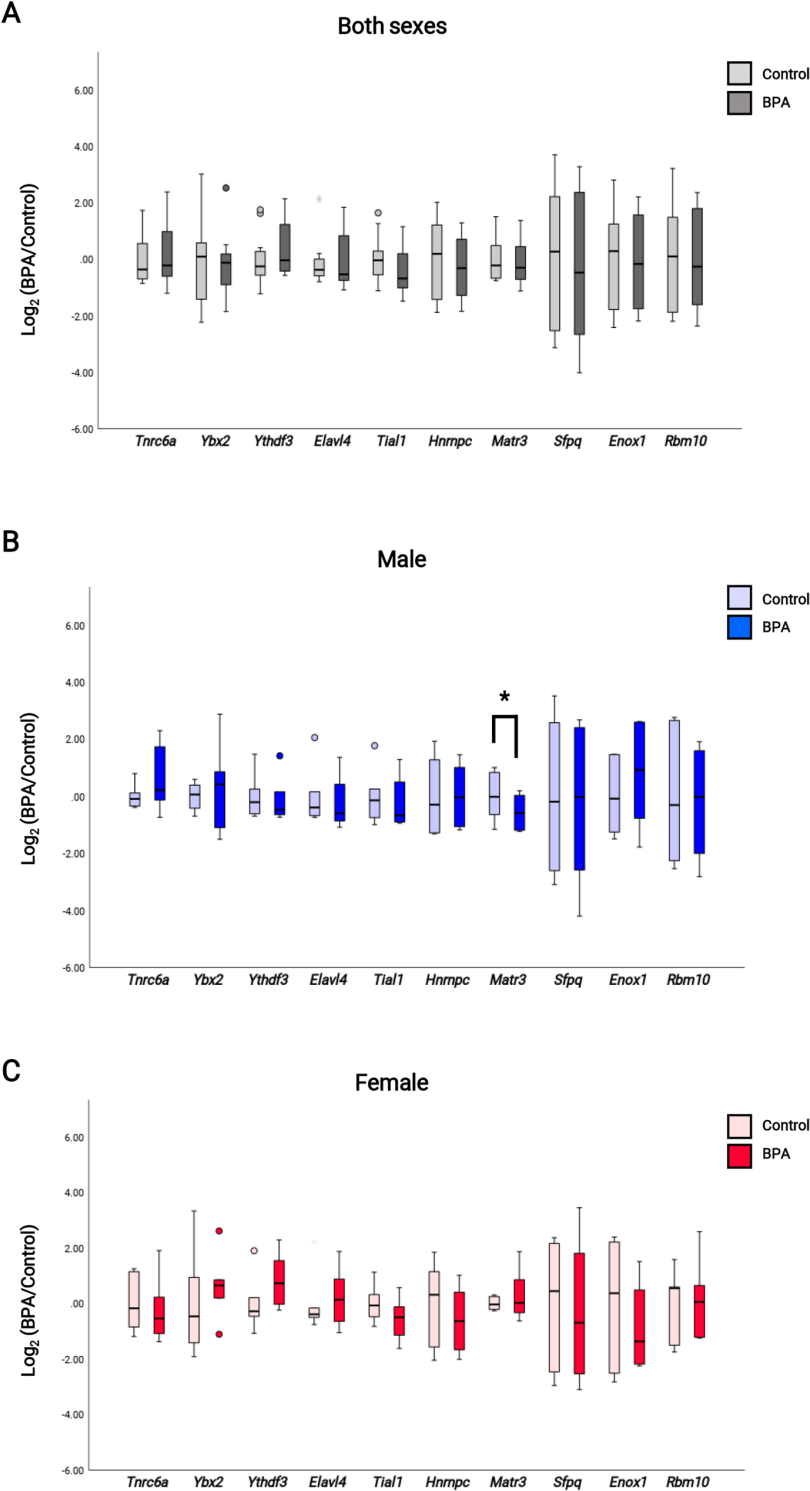
Boxplot of the RBPs expression analysis in the hippocampal tissues of rat offspring. (**A**) Quantitative analysis of expression levels of *Tnrc6a* (*p*-value = 0.555), *Ybx2* (*p*-value = 0.307), *Ythdf3* (*p*-value = 0.414), *Elavl4* (*p*-value = 0.995), *Tial1* (*p*-value = 0.336), *Hnrnpc* (*p*-value = 0.290), *Matr3* (*p*-value = 0.616), *Sfpq* (*p*-value = 0.446), *Enox1* (*p*-value = 0.893), *Rbm10* (*p*-value = 0.906) that were shown to have *Agap1* as their target in both sexes rat offspring prenatally exposed to BPA, (**B**) quantitative analysis of expression levels of *Tnrc6a* (*p*-value = 0.265), *Ybx2* (*p*-value = 0.623), *Ythdf3* (*p*-value = 0.834), *Elavl4* (*p*-value = 0.757), *Tial1* (*p*-value = 0.719), *Hnrnpc* (*p*-value = 0.943), *Matr3* (*p*-value = 0.005), *Sfpq* (*p*-value = 0.558), *Enox1* (*p*-value = 0.106), *Rbm10* (*p*-value = 0.631) that were shown to have *Agap1* as their target in male rat offspring prenatally exposed to BPA, (**C**) quantitative analysis of expression levels of *Tnrc6a* (*p*-value = 0.466), *Ybx2* (*p*-value = 0.406), *Ythdf3* (*p*-value = 0.222), *Elavl4* (*p*-value = 0.787), *Tial1* (*p*-value = 0.348), *Hnrnpc* (*p*-value = 0.193), *Matr3* (*p*-value = 0.531), *Sfpq* (*p*-value = 0.657), *Enox1* (*p*-value = 0.187), *Rbm10* (*p*-value = 0.815) that were shown to have *Agap1* as their target in female rat offspring prenatally exposed to BPA, * *p*-value < 0.05, paired t-test

We sought to identify which RBPs targeted our DAS genes and to understand how these RBPs were linked to BPA exposure. We hypothesized that BPA interacts with some transcription factors that target those RBPs. From previous studies, BPA has been known to act as a xenoestrogen and can interact with other transcription factors, such as androgen receptors (ARs), estrogen receptors (ERs), Yin Yang 1 (YY1), thyroid hormone receptors (THRs), and glucocorticoid receptors (GRs), among others [57, 69]. This study focused on the relationship between a BPA-related transcription factor and BPA-responsive differentially expressed genes (DEGs) identified by Thongkorn et al. [56, 57], as well as RBPs from oRNAment and POSTAR2 (Fig. 6). To determine whether the DEGs targeted by transcription factors (AR, ESR1, SOX5, and YY1) were functioning as RBPs, we performed a hypergeometric distribution analysis between targeted DEGs and RBPs. Interestingly, when we overlapped targeted DEGs with RBPs, we found that DEGs targeted by YY1 were significantly associated with RBPs in males (*p*-value = 3.63E-15) and females (*p*-value = 8.74E-19), for example, *Hnrnpc*, *Enox1*, *Tial1*, *Sfpq*, *Elavl4*, *Matr3*, and *Rbm5* (Table 4). This finding suggested that BPA exposure changes the expression of these RBPs through BPA-related transcription factors. Subsequently, we aimed to determine whether these RBPs targeted our DAS genes. Our DAS genes were then overlapped with the targeted genes of these RBPs using hypergeometric distribution analysis. Interestingly, our DAS from RNA-seq results were targeted by many RBPs associated with BPA-related transcription factors, as shown in Table 4. Among our DAS genes, *Agap1*, confirmed by HRM analysis, was the target of several RBPs that were controlled by the YY1 transcription factor, such as *Matr3* and *Hnrnpc*. Thus, we confirmed the expression analysis of these RBPs that target *Agap1*, including *Matr3*, *Hnrnpc*, and other RBPs from the list of other transcription factors. The expression analysis of RBPs showed that BPA exposure downregulated *Matr3* in the hippocampi of male rat offspring (Fig. 7A, B, and C). These results suggested that prenatal BPA exposure dysregulated the expression of alternative splicing regulators (the RBPs) through transcription factor YY1 in a sex-dependent manner, leading to changes in the alternative splicing process of DAS, particularly in the hippocampi of male rat offspring.

**Table 4:** Hypergeometric distribution of overlapping between BPA-associated TF targets, DEGs, RBPs, and DAS of rat offspring

Male												
Transcription factor	Number of transcription factor targets	Transcription factor target overlapped with DEGs (A)		Transcription factor target overlapped with RBPs (B)		DEGs overlapped with RPBs (C)			The number of targets from oRNAment and POSTAR2 overlapped with number of DEG	DAS (D)		
		Number of DEG	Hypergeometric distribution *p*-value	Number of RBPs	Hypergeometric distribution *p*-value	Number of DEGs and RBPs	Hypergeometric distribution *p*-value	Name		Number of DAS and RBP targets	Hypergeometric distribution *p*-value	Gene name
AR	755	88	2.22E-04	24	1.77E-02	7	1.79E-10	*Tial1*	3,760	9	2.37E-05	*Hnrnpr*,* Ubap2l*,* Ap2b1*,* Kif1b*,* Arl13b*,* Sh3kbp1*,* Kifap3*,* Lrp10*,* Znf689*
								*Tnrc6a*	700	2	6.56E-02	*Ap2b1*,* Ubap2l*
								*Tnrc6c*	95	1	6.51E-02	*Ap2b1*
								*Ythdf3*	2,925	8	4.20E-05	*Kif1b*,* Lrp10*,* Ap2b1*,* C19orf25*,* Ubap2l*,* Hnrnpr*,* Arl13b*,* Znf689*
ESR1	465	59	2.86E-04	12	2.20E-01	3	3.07E-05	*Tial1*	3,760	9	2.05E-05	*Hnrnpr*,* Ubap2l*,* Ap2b1*,* Kif1b*,* Arl13b*,* Sh3kbp1*,* Kifap3*,* Lrp10*,* Znf689*
								*Ythdf3*	2,925	8	4.02E-05	*Kif1b*,* Lrp10*,* Ap2b1*,* C19orf25*,* Ubap2l*,* Hnrnpr*,* Arl13b*,* Znf689*
SOX5	258	45	2.74E-06	8	1.51E-01	3	3.88E-06	*Tial1*	3,760	9	2.15E-05	*Hnrnpr*,* Ubap2l*,* Ap2b1*,* Kif1b*,* Arl13b*,* Sh3kbp1*,* Kifap3*,* Lrp10*,* Znf689*
								*Pum1*	4,364	7	7.89E-03	*Kif1b*,* Ap2b1*,* Arl13b*,* Lrp10*,* Ubap2l*,* Lypd6b*
YY1	744	97	3.15E-06	43	3.22E-09	11	3.63E-15	*Hnrnpc*	4,796	6	5.26E-02	*Kif1b*,* Kifap3*,* Arl13b*,* Ubap2l*,* Lypd6b*
								*Tial1*	3,760	9	2.26E-05	*Hnrnpr*,* Ubap2l*,* Ap2b1*,* Kif1b*,* Arl13b*,* Sh3kbp1*,* Kifap3*,* Lrp10*,* Znf689*
								*Pum1*	4,364	7	8.11E-03	*Kif1b*,* Ap2b1*,* Arl13b*,* Lrp10*,* Ubap2l*,* Lypd6b*
								*Sfpq*	4,882	8	2.87E-03	*Kif1b*,* Ap2b1*,* Kifap3*,* Arl13b*,* Lrp10*,* Ubap2l*,* Lypd6b*
								*Rbm10*	1,034	1	4.76E-01	*Hnrnpr*
								*Rbm5*	5,004	7	1.67E-02	*Kif1b*,* Ap2b1*,* Kifap3*,* Rhot1*,* Lrp10*,* Ubap2l*,* Lypd6b*

Female												
Transcription factor	Number of transcription factor targets	Transcription factor target overlapped with DEGs (A)		Transcription factor target overlapped with RBPs (B)		DEGs overlapped with RPBs (C)			The number of targets from oRNAment and POSTAR2 overlapped with number of DEG	DAS (D)		
		Number of DEG	Hypergeometric distribution *p*-value	Number of RBPs	Hypergeometric distribution *p*-value	Number of DEGs and RBPs	Hypergeometric distribution *p*-value	Name		Number of DAS and RBP targets	Hypergeometric distribution *p*-value	Gene name
AR	755	124	8.77E-03	24	1.56E-02	7	2.56E-09	*Elavl4*	4,460	15	2.07E-05	*Kif1b*,* Hnrnpc*,* Ints10*,* Mtg2*,* Kctd17 Arl13b*,* Hnrnpdl*,* Abt1*,* Meis2*,* Ubxn2b*,* Maf1*,* Agap1*,* Ankrd10*,* Ubap2l*,* Pik3cd*,* Rhot1*
								*Hnrnpd*	3,437	12	6.43E-05	*Slc19a1*,* Kif1b*,* Hnrnpc*,* Ints10*,* Arl13b*,* Abt1*,* Meis2*,* Ubxn2b*,* Cyp20a1*,* Ankrd10*,* Ubap2l*,* Pik3cd*,* Rhot1*
								*Tial1*	3,767	14	2.78E-05	*Hnrnpc*,* Hnrnpdl*,* Ankrd10*,* Ubap2l*,* Ints10*,* Kif1b*,* Rhot1*,* Slc15a4*,* Agap1*,* Maf1*,* Cyp20a1*,* Meis2*,* Slc19a1*,* Ubxn2b*
								*Tnrc6a*	2,925	8	4.67E-02	*Slc19a1*,* Agap1*,* Ubap2l*
ESR1	465	82	5.94E-03	12	2.03E-01	4	2.01E-06	*Elavl4*	4,640	15	2.42E-05	*Kif1b*,* Hnrnpc*,* Ints10*,* Mtg2*,* Kctd17 Arl13b*,* Hnrnpdl*,* Abt1*,* Meis2*,* Ubxn2b*,* Maf1*,* Agap1*,* Ankrd10*,* Ubap2l*,* Pik3cd*,* Rhot1*
								*Tial1*	3,767	14	2.45E-05	*Hnrnpc*,* Hnrnpdl*,* Ankrd10*,* Ubap2l*,* Ints10*,* Kif1b*,* Rhot1*,* Slc15a4*,* Agap1*,* Maf1*,* Cyp20a1*,* Meis2*,* Slc19a1*,* Ubxn2b*
								*Ybx2*	2,378	9	4.95E-04	*Kif1b*,* Slc15a4*,* Hnrnpc*,* Hnrnpdl*,* Meis2*,* Agap1*,* Ankrd10*,* Ubap2l*,* Pik3cd*
SOX5	258	58	7.18E-05	11	1.56E-02	2	8.06E-04	*Tial1*	3,767	14	2.60E-05	*Hnrnpc*,* Hnrnpdl*,* Ankrd10*,* Ubap2l*,* Ints10*,* Kif1b*,* Rhot1*,* Slc15a4*,* Agap1*,* Maf1*,* Cyp20a1*,* Meis2*,* Slc19a1*,* Ubxn2b*
								*Elavl4*	4,640	15	2.90E-05	*Kif1b*,* Hnrnpc*,* Ints10*,* Mtg2*,* Kctd17 Arl13b*,* Hnrnpdl*,* Abt1*,* Meis2*,* Ubxn2b*,* Maf1*,* Agap1*,* Ankrd10*,* Ubap2l*,* Pik3cd*,* Rhot1*,
YY1	744	140	2.51E-05	43	3.35E-09	14	8.74E-19	*Hnrnpc*	4,832	14	6.66E-05	*Kif1b*,* Slc15a4*,* Hnrnpc*,* Ints10*,* Mtg2*,* Arl13b*,* Hnrnpdl*,* Meis2*,* Ubxn2b*,* Maf1*,* Cyp20a1*,* Agap1*,* Ankrd10*,* Ubap2l*,* Rhot1*
								*Enox1*	3,379	9	5.48E-03	*Kif1b*,* Slc15a4*,* Ints10*,* Mtg2*,* Meis2*,* Maf1*,* Agap1*,* Ankrd10*,* Ubap2l*,* Rhot1*
								*Tial1*	3,767	14	2.31E-05	*Hnrnpc*,* Hnrnpdl*,* Ankrd10*,* Ubap2l*,* Ints10*,* Kif1b*,* Rhot1*,* Slc15a4*,* Agap1*,* Maf1*,* Cyp20a1*,* Meis2*,* Slc19a1*,* Ubxn2b*
								*Sfpq*	4,869	12	1.55E-03	*Slc19a1*,* Kif1b*,* Hnrnpc*,* Ints10*,* Kctd17*,* Arl13b*,* Abt1*,* Ubxn2b*,* Cyp20a1*,* Agap1*,* Ubap2l*,* Pik3cd*,* Rhot1*
								*Elavl4*	4,640	15	1.81E-05	*Kif1b*,* Hnrnpc*,* Ints10*,* Mtg2*,* Kctd17 Arl13b*,* Hnrnpdl*,* Abt1*,* Meis2*,* Ubxn2b*,* Maf1*,* Agap1*,* Ankrd10*,* Ubap2l*,* Pik3cd*,* Rhot1*
								*Rbm10*	1,045	3	1.15E-01	*Hnrnpdl*,* Agap1*,* Rhot1*
								*Matr3*	1,678	5	2.92E-02	*Kif1b*,* Hnrnpc*,* Cyp20a1*,* Agap1*,* Ubap2l*,* Rhot1*
								*Rbm5*	4,996	15	2.80E-05	*Slc19a1*,* Ogfr*,* Kif1b*,* Slc15a4*,* Hnrnpc*,* Ints10*,* Mtg2*,* Kctd17*,* Abt1*,* Meis2*,* Ubxn2b*,* Cyp20a1*,*Ankrd10*,* Ubap2l*,* Rhot1*

(A) The number of transcription factor targets overlapped with DEGs, (B) the number of transcription factor targets overlapped with RBPs, (C) the number of DEGs overlapped with RBPs, and (D) the number of BPA-responsive RBPs overlapped with DAS

### BPA exposure decreased YY1 binding at the promoter of *Matr3*

**Fig. 8:**
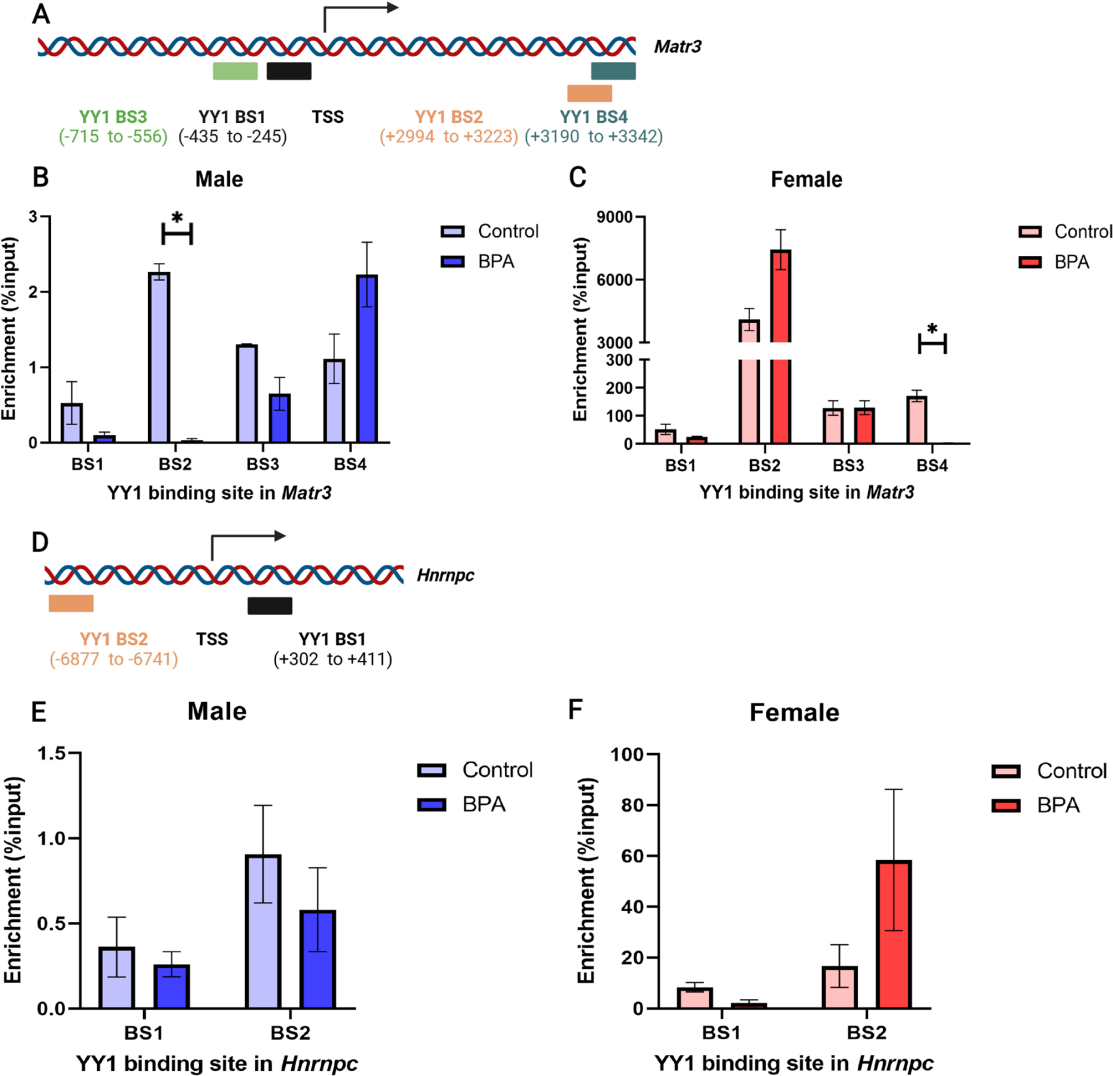
BPA disrupted YY1 binding at *Matr3* and *Hnrnpc* promoters in male and female rat offspring. (**A**) Schematic diagram of YY1 binding sites at promoter of *Matr3* created in https://BioRender.com, (**B**, **C**) YY1 enrichment at the binding site of *Matr3* in male and female rat offspring, respectively (male BS1 *p*-value = 0.839, BS2 *p*-value = 0.010, BS3 *p*-value = 0.193, BS4 *p*-value = 0.651, female BS1 *p*-value = 0.335, BS2 *p*-value = 0.068, BS3 *p*-value = 0.175, BS4 *p*-value = 0.032), (**D**) Schematic diagram of YY1 binding sites at promoter of *Hnrnpc* created in https://BioRender.com, (**E**, **F**) YY1 enrichment at the binding sites of *Hnrnpc* in male and female rat offspring, respectively (male BS1 *p*-value = 0.058, BS2 *p*-value = 0.162, female BS1 *p*-value = 0.236, BS2 *p*-value = 0.228), *p-value < 0.05, paired t-test

The ChIP-qPCR analysis of YY1 enrichment at the promoter of *Matr3* and *Hnrnpc*, which were splicing regulators of *Agap1*, revealed that YY1 binding was significantly decreased at the promoter binding site number 2 (BS2) of *Matr3* in male rat offspring prenatally exposed to BPA compared with the vehicle control group. Moreover, YY1 binding also decreased at promoter binding site 4 (BS4) of *Matr3* in female rat offspring prenatally exposed to BPA compared to the vehicle control (Fig. 8B and C). However, we observed no significant changes in the enrichment of the two promoter binding sites of *Hnrnpc*, both males and females (Fig. 8E and F). These results indicated that BPA dysregulated YY1 binding at the promoter of *Matr3*, leading to the downregulation of *Matr3* expression in a sex-dependent manner, which caused differential alternative splicing of *Agap1* in males.

## Discussion

Previous studies have reported the effects of BPA on alternative splicing in the blood, kidney, and testis [26, 55, 70]. Although our recent studies have shown that prenatal BPA exposure resulted in the dysregulation of genes associated with ASD in the prefrontal cortex and hippocampus of rat offspring, the effects of prenatal BPA exposure on alternative splicing of genes in the hippocampus have not been investigated. We are the first to demonstrate that prenatal BPA exposure disrupts YY1 binding at the promoter of *Matr3* and alters *Agap1* splicing in the hippocampus of rat offspring in a male-specific manner.

In our study, we selected a BPA dose of 5,000 µg/kg of maternal body weight/day, which corresponds to the No-Observed-Adverse-Effect Level (NOAEL) established by the European Food Safety Authority (EFSA) and the U.S. Food and Drug Administration (FDA) [71, 72]. This dose was chosen to investigate the subtle molecular changes induced by BPA exposure while avoiding overt toxicity that may confound interpretation. While previous studies have demonstrated that high doses of BPA—ranging from 100 to 300 mg/kg/day—can cause evident hormonal disruptions, such as impaired ovarian function in mice [73] and decreased levels of testosterone and estradiol in rats [74], our findings revealed that the NOAEL dose of BPA could cause dysregulation of alternative splicing in the hippocampus of offspring. Notably, earlier studies have shown that BPA exposure at doses above 20 mg/kg/day can upregulate genes encoding splicing regulators, such as U5 snRNA and *Hnrnpu*, particularly in the testis [75], and also reduce newly generated hippocampal cells and significantly impair the learning and memory of mice [76]. Moreover, prenatal BPA exposure revealed differential alternatively spliced of genes in the prefrontal cortex of rat offspring, leading to ASD-related behaviors including anxiety, hyperactivity, and aggression [77]. These observations support our findings, as we demonstrate that prenatal BPA exposure at the NOAEL dose is sufficient to induce specific molecular alterations in the developing hippocampus, particularly by disrupting the regulation of alternative splicing.

Several studies have demonstrated that BPA exposure can impact multiple biological pathways, including the dysregulation of gene expression, alterations in alternative splicing, and disruption of neurodevelopmental and behavioral processes. Our previous studies reported that prenatal BPA exposure disrupted the transcriptome profile of neonatal rat offspring, particularly in the hippocampus [56]. Moreover, several ASD-related genes (e.g., *Mief2*, *Eif3h*, *Cux1*, *Atp8a1*) exhibited sex-specific dysregulation, with a male bias, and were associated with neuronal viability, neuritogenesis, and learning/memory [78]. Human studies have also reported sex-specific behavioral effects of BPA exposure linked to hippocampal function. For example, Harley et al. (2013) found that higher prenatal BPA levels were associated with increased internalizing problems (e.g., anxiety and depression) in boys at age 7, while no associations were seen in girls [79]. Similarly, elevated prenatal BPA exposure was linked to increased behavioral problems in boys, including heightened emotional reactivity and aggressive behavior [80]. Further, BPA exposure has been shown to affect alternative splicing. One study revealed that BPA-exposed male rats exhibited increased depressive-like behaviors alongside reductions in signaling pathways related to mineralocorticoid receptor-induced nNOS activation [81], aligning with previous findings that individuals with ASD are at increased risk of co-occurring depression [82]. A genome-wide study in individuals with ASD reported downregulation of neuron-specific exons and diminished gene expression differences between the frontal and temporal lobes, potentially involving the transcription factor SOX5 [27]. Moreover, mRNA analysis from the blood of young boys with ASD identified 53 genes with predicted differential alternative splicing compared to typically developing individuals, including splicing regulators and genes involved in ASD-associated pathways [26]. Additional studies have shown that the aging brain exhibits altered splicing of methionine synthesis-related mRNAs, with ASD brains showing a more rapid decline in alternative splicing [25]. Collectively, these findings highlight a potential mechanism by which BPA exposure contributes to the male bias observed in ASD prevalence. Previous research also identified DAS in the kidney of BPA-exposed mice, involving components of Wnt signaling [70], and reduced spliceosome activity in the testis during adolescence [55]. However, no studies had examined the effects of prenatal BPA exposure on alternative splicing in the hippocampus. In this study, we reveal for the first time that ASD-related genes exhibit differential alternative splicing patterns in the hippocampus of rat offspring prenatally exposed to BPA, with notable sex-specific differences.

We found that prenatal BPA exposure caused an increase in alternative events of *Agap1*, which is associated with ASD. *Agap1* (Arf-GAP with GTPase, ANK repeat and PH domain-containing protein 1) is gene encoded protein Arf1-Gap with GTPase, ANK repeat and PH domain-containing protein 1 that activates a protein that interacts with vesicle complexes adaptor protein 3 (AP-3) [83] and Biogenesis of Lysosome Related Organelles Complex-1 (BLOC-1) [84]. A genotyping study revealed a variant at the AGAP1 locus on chromosome 2q37.3 in a proband of ASD. They identified Ser > Gly substitutions and Arg > Gly substitutions, which are linked to the region of ASD susceptibility. The study also revealed that *Agap1* is localized in axons and dendrites, thereby affecting the trafficking of neurons involved in the morphogenesis of the synapse [85]. We also put the alternatively spliced product sequence into the NCBI Conserved Domain Database (https://www.ncbi.nlm.nih.gov/Structure/cdd/wrpsb.cgi) [86] and found that the alternative product of this gene was in the PH domain of the protein. The PH domain is involved in targeting proteins to their cellular location or interacting with their partners. This result suggested that prenatal BPA exposure dysregulated alternative splice events in *Agap1*, especially this skipped exon corresponding to the PH1-like domain, which might affect neuronal functions. Although BPA exposure did not alter the total expression of *Agap1*, changes in alternative splicing may still influence protein structure and function. Alternative splicing generates isoforms with varying stability, interactions, and localization, leading to functional differences despite unchanged overall gene expression [51]. Indeed, a meta-analysis of RNA-seq data demonstrated that differentially expressed and differentially spliced genes regulate distinct biological processes [87]. In this study, qRT-PCR measured total gene expression across all transcript variants, not specific isoforms, highlighting that isoform-level changes can have significant biological consequences. Moreover, prenatal BPA exposure also disrupts the alternative splicing of *Ap2b1* (Adaptor-related protein complex 2 subunit beta1), which encodes an adaptor protein involved in cell trafficking, especially in neurons [88]. The AP-2 complex plays a crucial role in neurons by regulating AMPARs and NMDARs [89, 90]. *AP2B1* upregulation disrupts synaptic plasticity, leading to neuronal and cognitive dysfunctions observed in Fragile X syndrome [91]. Furthermore, qPCR-HRM analysis revealed that prenatal BPA exposure also disrupts the alternative splicing of *Kifap3* (Kinesin-associated protein 3). This gene encodes a protein involved in kinesin function during intracellular transport. It affects neurological diseases, especially amyotrophic lateral sclerosis, by modulating intracellular transport, which contributes to neurodegeneration [92]. A genome-wide association study in cattle revealed an overlap between the differential expression of this gene in the human brain and pituitary gland and in cattle. It plays a crucial role in neuronal migration, which can impact brain development [93]. However, there is no direct evidence investigating the molecular mechanisms of AP2B1 and KIFAP3 in ASD. We are the first to demonstrate that prenatal BPA exposure disrupts the alternative splicing of these genes, which play essential roles in neurons, potentially affecting brain development and contributing to the causes of ASD.

Alternative splicing is a key mechanism for regulating gene expression, enhancing transcript and protein diversity, and guiding tissue development. It is governed by cis-acting elements, trans-acting RNA-binding proteins (RBPs), and the dynamic interplay between transcription and splicing. Factors such as chromatin structure and RNA conformation also contribute to splicing outcomes [51]. Han et al. (2017) predicted hundreds of transcription factors involved in splicing regulation using SPAR-seq, with one-third confirmed to directly or indirectly influence splicing [94]. Another regulator of alternative splicing is the RBPs, which act as trans-acting factors by binding to SREs in regulating alternative splicing [52].

To investigate how BPA-related transcription factors might influence splicing, we referenced our previous study [57], which identified BPA-responsive transcription factors. Among these, YY1 emerged as a key regulator. YY1, one of the BPA-related transcription factors, is a transcriptional protein. It plays a role in both repressing and activating a variety of gene promoters, indicating its dual functionality [95]. YY1 can recruit histone deacetylases and acetyltransferases to influence promoter activity, affecting histone modifications. YY1 has demonstrated sex-specific regulatory roles; for example, overexpression of YY1 increased left ventricular size in both sexes, but only induced cardiac dysfunction in males, suggesting dimorphic regulatory potential [96]. The YY1 protein consists of four zinc-finger motifs and has specific domains for transcriptional activation and repression. YY1’s regulatory function is characterized by its ability to switch between activation and repression of transcription, depending on the presence of other proteins [97]. While Thongkorn et al. (2023) demonstrated that BPA can bind YY1 via molecular docking, the downstream effects on activation or repression remain unclear.

In our study, YY1 binding activity was reduced at the *Matr3* promoter only in male offspring exposed to BPA, leading to decreased *Matr3* expression. *Matr3*, along with PTBP, is known to repress exon inclusion and stabilize chromatin architecture through interactions with YY1 [98, 99]. *Matr3* also plays a specific role with YY1 to stabilize the chromatin structure and organize the transcription with YY1. Loss of *Matr3* has been associated with increased cryptic exon inclusion, contributing to neurodegenerative diseases [100]. RNA-seq data revealed that BPA did not change YY1 expression at the transcript level in either sex, suggesting that BPA affects YY1 activity through post-translational mechanisms or signaling cascades that alter its binding and chromatin remodeling functions. This dimorphic reduction in YY1 activity resulted in downregulation of *Matr3* in males, thereby impairing its regulatory role in splicing. The increased alternative splicing of *Agap1* in males aligns with our previous findings that male offspring are more susceptible to BPA-induced transcriptomic disruptions in the hippocampus, which are associated with impaired learning and memory [56, 78]. These findings highlight a novel pathway by which prenatal BPA exposure may contribute to ASD pathogenesis through sex-specific disruption of YY1-*Matr3*-mediated splicing regulation. While this study offers valuable insights, it also underscores the need for further investigation into the mechanisms by which BPA and YY1 influence alternative splicing and behavior.

Previous studies revealed that BPA induced the differential expression of genes linked to ASD, including candidates *Auts2* and *Foxp2* [56]. Sex-specific effects of BPA have also been observed, mediated through transcription factors like AR, ESR1, and RORA in the prefrontal cortex of rat offspring [46]. In this study, we performed an interactome analysis of DAS genes using IPA software. We found that DAS genes were associated with several biological functions/diseases in a sex-dependent manner. In males, BPA-responsive DAS genes such as *Hnrnpr*, *Kif1b*, and *Celf1* were enriched in pathways related to alternative mRNA splicing and nervous system differentiation [101–103]. In contrast, in females, DAS gene networks, including *Ubap2l*, *Pik3cd*, and *Meis2*, were primarily associated with neuritogenesis, neuronal development, and intellectual disability [104–106]. These results raise concerns about the safety of BPA exposure during pregnancy and its role as a potential risk factor for ASD.

Limitations of our study include the relatively small sample size. RNA-seq analysis was performed on six pups per treatment group (three males and three females), while validation by qPCR-HRM was conducted on an independent cohort of 12 animals per group (six males and six females). Future studies should aim to increase the sample size to enhance statistical power. Additionally, HRM analysis was limited in detecting specific splice variants due to technical challenges. For example, *Kif1b*’s mutually exclusive exons had identical sequences, making them indistinguishable by HRM or gel electrophoresis. *Slc19a1*’s intron retention occurred in the untranslated region, preventing efficient PCR amplification [107]. *Meis2*’s alternative 3’ splice site was influenced by various factors, such as temperature, RNA structure, and intronic context [108–110], complicating the detection of splice changes. These limitations underscore the need for more comprehensive approaches, such as full-length transcriptome sequencing, to characterize splicing variants better. Finally, future research should incorporate human-based models such as neuronal cell cultures or brain organoids to replicate human neurodevelopment and alternative splicing regulation better. These models could provide deeper insights into the impact of BPA on splicing mechanisms and its contribution to ASD susceptibility in humans.

## Conclusion

**Fig. 9:**
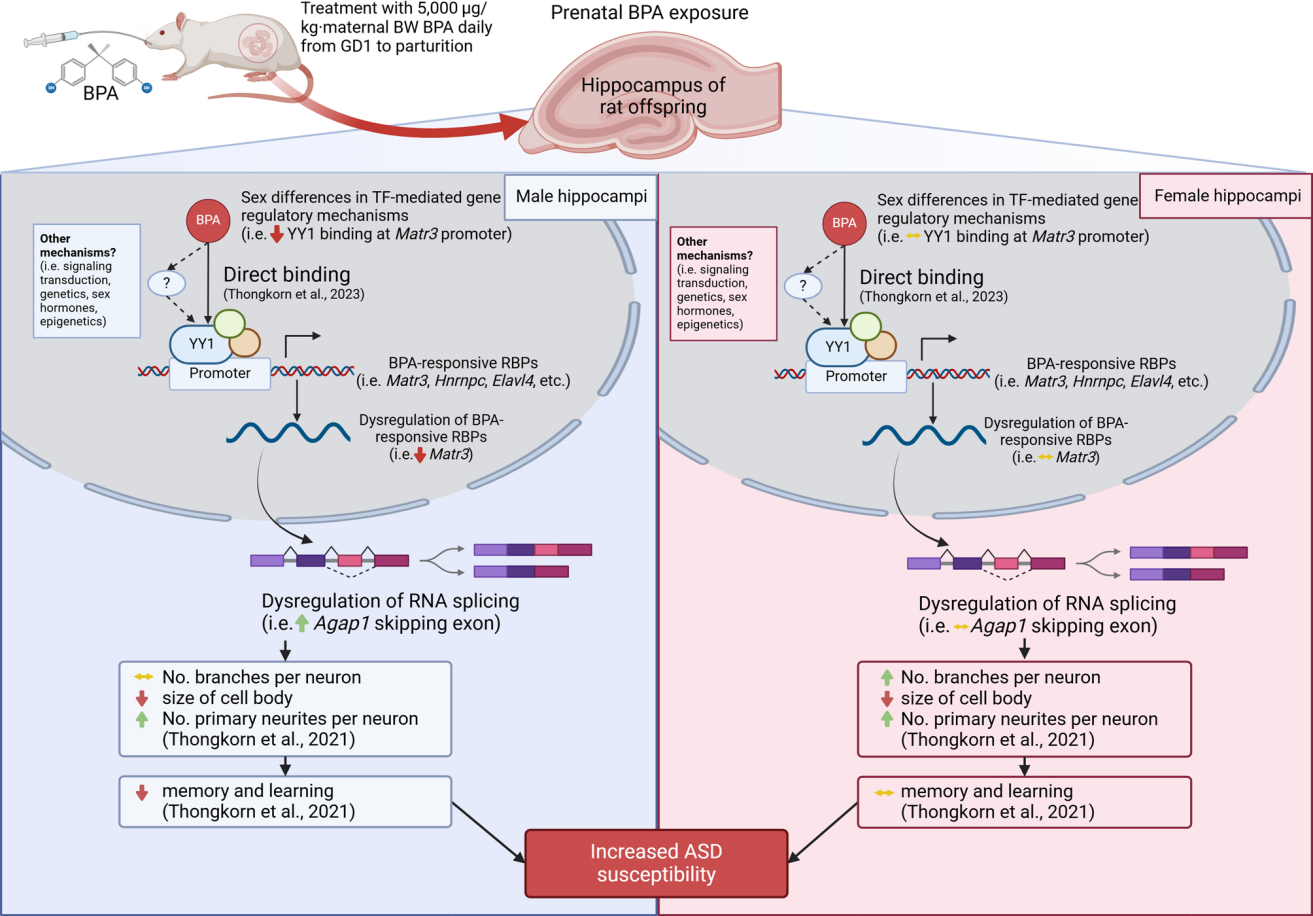
A schematic diagram illustrating the potential mechanisms of prenatal BPA effects on alternative splicing in the hippocampal tissues in male and female rat offspring. This figure was created in https://BioRender.com

This study provides suggestive evidence that prenatal BPA exposure disrupts alternative splicing of ASD-related genes in a sex-dependent manner through transcription factor-mediated regulation of splicing factors. RNA-seq analysis revealed that BPA exposure altered the alternative splicing of *Agap1*, a gene associated with ASD, without changing its overall expression level. qPCR-HRM analysis further confirmed an increase in the alternatively spliced *Agap1* transcript, specifically in the hippocampus of male rat offspring. Functional analysis of BPA-responsive DAS genes indicated enrichment in pathways related to neurological, developmental, and psychiatric disorders, including synaptogenesis, neuritogenesis, intellectual disability, and synapse formation—biological processes commonly affected in ASD. Notably, these networks also highlighted mRNA splicing regulation, emphasizing its potential as a key mechanism underlying BPA-induced neurodevelopmental disruption. ChIP-qPCR results demonstrated sex-specific differences in YY1 transcription factor binding at the *Matr3* promoter, with decreased binding observed in males. This reduction in YY1 enrichment corresponded with the downregulation of *Matr3* expression and an increase in *Agap1* alternative splicing events, particularly in male offspring. These findings align with our hypothesis that BPA exposure impairs splicing regulation via transcription factor dysregulation in a male-biased manner, contributing to ASD susceptibility. Together, our results suggest that prenatal BPA exposure interferes with the transcription factor-mediated regulation of splicing, especially in males, resulting in altered alternative splicing patterns of critical neurodevelopmental genes such as *Agap1* (Fig. 9). These molecular alterations may underlie the sex-biased behavioral and developmental features observed in ASD, raising further concerns about the neurodevelopmental risks of BPA exposure during pregnancy.

## Supplementary Information

Below is the link to the electronic supplementary material.

Supplementary Material 1: Additional file 1 The list of primers for qPCR-HRM, qRT-PCR, and ChIP-qPCR analyses

Supplementary Material 2: Additional file 2 All detectable splicing events in the hippocampus of male rat offspring

Supplementary Material 3: Additional file 3 All detectable splicing events in the hippocampus of female rat offspring

Supplementary Material 4: Figure 1 High-resolution melting analysis of *Cyp20a1* isoform. Figure 2 Validation of the sequences of the qPCR-HRM products of *Agap1*. Figure 3 Validation of the sequences of the qPCR-HRM products of *Ap2b1*. Figure 4 Validation of the sequences of the qPCR-HRM products of *Kifap3*

## Data Availability

The dataset used in this study is available in the NCBI GEO Datasets repository under accession number GSE140298 (https://www.ncbi.nlm.nih.gov/geo/query/acc.cgi?acc=GSE140298).
