# Supplementary material for: Sex-dependent epigenetic disruption of YY1 binding by prenatal BPA exposure downregulates Matr3 and alters Agap1 splicing in the offspring hippocampus
Source: Biol Sex Differ. 2025 Aug 11;16:63. doi: 10.1186/s13293-025-00744-1 (PMC12337383; doi:10.1186/s13293-025-00744-1)
Supplement: Supplementary file 1 — Supplementary Material 1: Additional file 1 The list of primers for qPCR-HRM, qRT-PCR, and ChIP-qPCR analyses [file 13293_2025_744_MOESM1_ESM.docx]

Supplementary table 1: Primer for qRT-PCR-HRM analysis

| Primer name | Sequences (5’->3’) |
| --- | --- |
| F_Agap1 | GAGCAGTTTACACATCTCACCA |
| R_Agap1 | GCTTCAAAGTGCCACGTCTG |
| F_Ap2b1 | GAACAGCCTCAGGTCATCCC |
| R_Ap2b1 | GGCACTGATGACGGGATGAA |
| F_Cyp20a1 | CCAGGGCTTACTCCAACTGA |
| R_Cyp20a1 | ACATGGTTTCAAATGGGTCCA |
| F_Kif1b | GTTCGAGTGAGGCCCTTCAA |
| R_Kif1b | ATTGTACACGCGGCTCTGAG |
| F_Kifap3 | GAAGGCTCTTGATCGGGACA |
| R_Kifap3 | TGGTAAAGCACACACATCGC |
| F_Meis2 | ACAGCCCATGATTGACCAGT |
| R_Meis2 | TGCCCATCCATGCCCATATT |
| F_Slc19a1 | TAGATGCCGCCTCCACACTT |
| R_Slc19a1 | CTCGCGATGACCAGCTTTGA |

Supplementary table 2: Primer for qRT-PCR in expression level analysis

| Primer name | Sequences (5’->3’) |
| --- | --- |
| F_Agap1 | CTGTGGGGAAGGAGATGGTC |
| R_Agap1 | AAGCACGTCGATACACTCCT |
| F_Ap2b1 | TGGGCCCAGTCATGAAGATG |
| R_Ap2b1 | GAATATCCTTCCACGTCGCA |
| F_Cyp2011 | CGTCCTTTATGCCCTCGGAG |
| R_Cyp20a1 | TGTGCCTGAGAAGCCAAGAG |
| F_Elavl4 | GTACGGTCGCATCATCACCT |
| R_Elalv4 | GACTTCTGGCTGGGGTTGTT |
| F_Enox1 | GACCGAGAAAACAACACCGA |
| R_Enox1 | TCCATCTCTTCTCCAGCGTC |
| F_Hnrnpc | GTGGATTCTCTGCTGGAAAGC |
| R_Hnrnpc | CACTGCTCTGCTCCTCTTCA |
| F_Kif1b | CCAGTAGAGCGTGGCATCAT |
| R_Kif1b | CAGGAGTGGGTTAAAGGCGT |
| F_Kifap3 | TCCCAGCACTCATCGAACTG |
| R_Kifap3 | ACGACTCTCCACCATCTCCA |
| F_Matr3 | GGTTATCCCCATCTGTGCTCT |
| R_Matr3 | GGTCCCAGAATTCCCGGTG |
| F_Meis2 | AGTTAGCACAAGACACGGGG |
| R_Meis2 | GGATCCCCATGTGTTGCTGA |
| F_Rbm10 | CCCCAAGCCCAAGATCAATG |
| R_Rbm10 | TGCTTCTGATTTGGGCACAC |
| F_Rn18s | CTGGATACCGCAGCTAGGAA |
| R_Rn18s | GAATTTCACCTCTAGCGGCG |
| F_Sfpq | TAGGTGGTGGTGGTGGCATA |
| R_Sfpq | TTCCAGGCCCCATTCCTCTA |
| F_Slc19a1 | GTCCATCATCTGCCTTGCCT |
| R_Slc19a1 | ATCGCCTGCACAGAATCCTC |
| F_Tial1 | AGCAACCCGATAGCAGAAGG |
| R_Tial1 | ACTTGGTGTTGTTGCCCAGT |
| F_Tnrc6a | CTCAGACCAGCAAGCACAGA |
| R_Tnrc6a | GTCGTGGCTGGAGTAGAAGG |
| F_Ybx2 | CAACCCCAGGAAGTTTCTGC |
| R_Ybx2 | GGCCGAGGAATGAATCTACG |
| F_Ythdf3 | CAGCCACTAGCGTGGATCAG |
| R_Ythdf3 | TCTGACATTGGTGGATAGCTGTT |

Supplementary table 3: Primer for ChIP-qPCR analysis

| Primer name | Sequences (5’->3’) |
| --- | --- |
| F_Matr3_BS1 | ACTCTTCCTTCCGCTCTTCC |
| R_Matr3_BS1 | GCGTTAGGTGGGACTCTCTA |
| F_Matr3_BS2 | ACAGAGGTCCTAATCAGATCAGA |
| R_Matr3_BS2 | CTCTGAGACTGAGGCTAGCC |
| F_Matr3_BS3 | TTTCCAGCCTCCCCTTATGT |
| R_Matr3_BS3 | CTCAGCCTCCCAGTTAGCAA |
| F_Matr3_BS4 | CTTGTGTAGATCAGGCTAGCC |
| R_Matr3_BS4 | CATTGTGGTTTCTTTCTGTTCCA |
| F_Hnrnpc_BS1 | CGGTGCGTACGAAAATACCC |
| R_Hnrnpc_BS1 | TCTTCGCTTTCTTGCTCCCC |
| F_Hnrnpc_BS2 | CTGGCCATTGGTTTTGCAGA |
| R_Hnrnpc_BS2 | GTGGCCTTGTCAGTGAACAG |
