# Supplementary material for: Sex-dependent epigenetic disruption of YY1 binding by prenatal BPA exposure downregulates Matr3 and alters Agap1 splicing in the offspring hippocampus
Source: Biol Sex Differ. 2025 Aug 11;16:63. doi: 10.1186/s13293-025-00744-1 (PMC12337383; doi:10.1186/s13293-025-00744-1)
Supplement: Supplementary file 4 — Supplementary Material 4: Figure 1 High-resolution melting analysis of Cyp20a1 isoform. Figure 2 Validation of the sequences of the qPCR-HRM products of Agap1. Figure 3 Validation of the sequences of the qPCR-HRM products of Ap2b1. Figure 4 Validation of the sequences of the qPCR-HRM products of Kifap3 [file 13293_2025_744_MOESM4_ESM.docx]

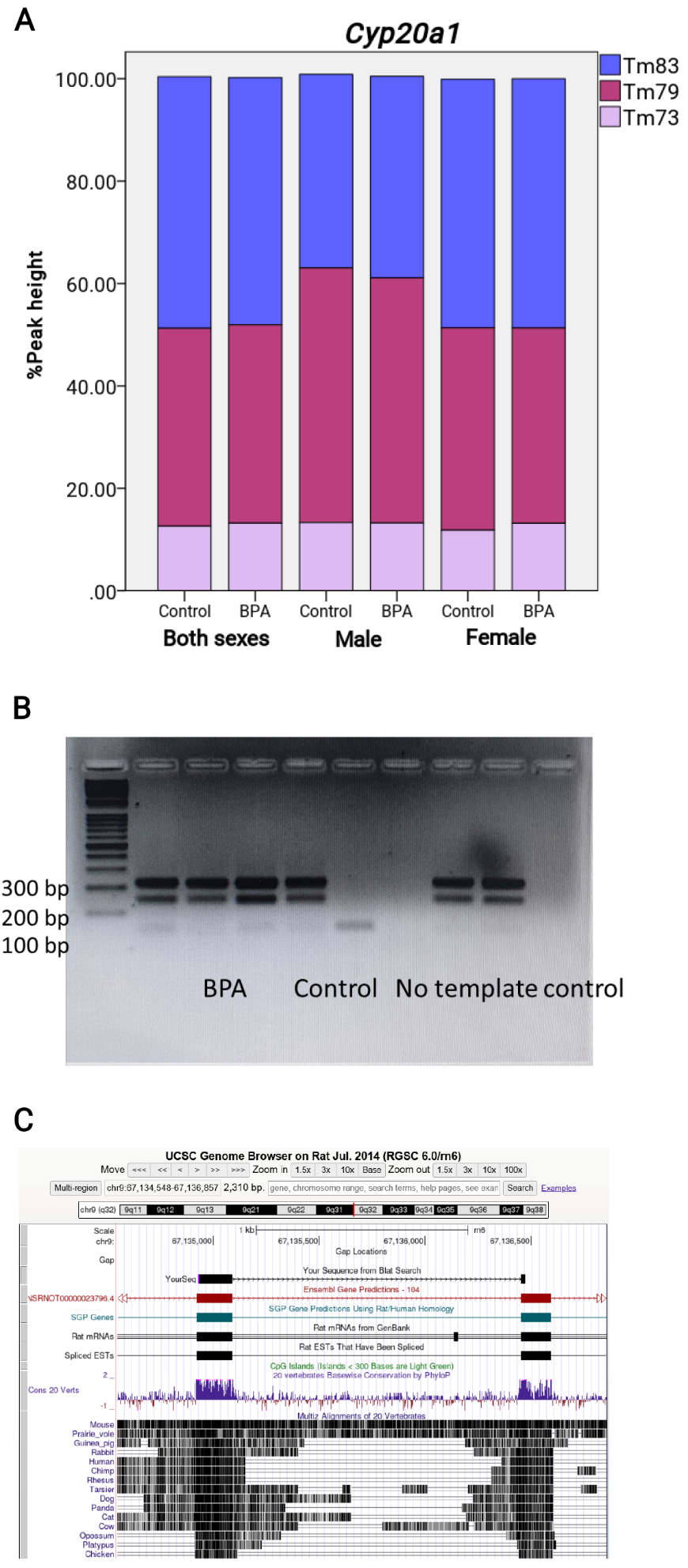


**Supplementary Figure 1 High-resolution melting analysis of *Cyp20a1* isoform**

(A) High-resolution melting analysis of *Cyp20a1* isoform using % peak height of each product (both sexes Tm 73°C p-value = 0.631, Tm 79°C p-value = 0.832, Tm83°C p-value = 0.685, male Tm 73°C p-value = 0.964, Tm 79°C p-value = 0.569, Tm 83°C p-value = 0.735, female Tm 73°C p-value = 0.532, Tm 78°C p-value = 0.596, Tm 83°C p-value = 0.864), (B) Gel electrophoresis of *Cyp20a1* showing multiple products. The first band from below was a 43 bp product, and the second band was a 246 bp product., (C) BLAT results of the 246 bp product of *Cyp20a1* sequencing from Sanger’s sequencing revealed the match sequences of the 246 bp product and the theoretical product of *Cyp20a1* in-silico PCR test.

**
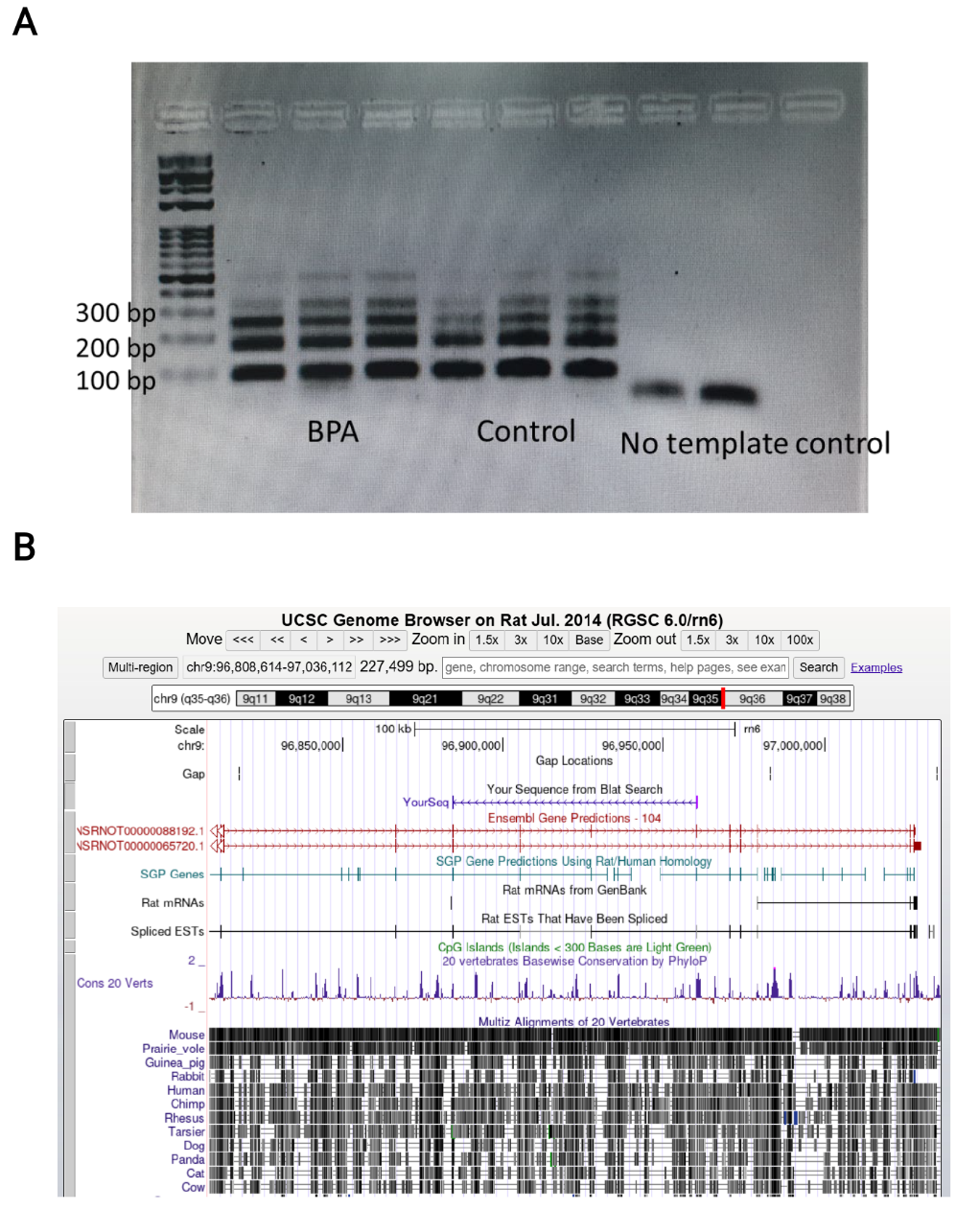
**

**Supplementary Figure 2 Validation of the sequences of the qPCR-HRM products of *Agap1***

(A) Gel electrophoresis of *Agap1* showing multiple products. The first band from below was 106 bp product, and the second band was 252 bp product., (B) BLAT results of the 252 bp of *Agap1* sequencing from Sanger’s sequencing revealed the match sequences of the 252 bp product and the theoretical product from the in-silico PCR test.

**
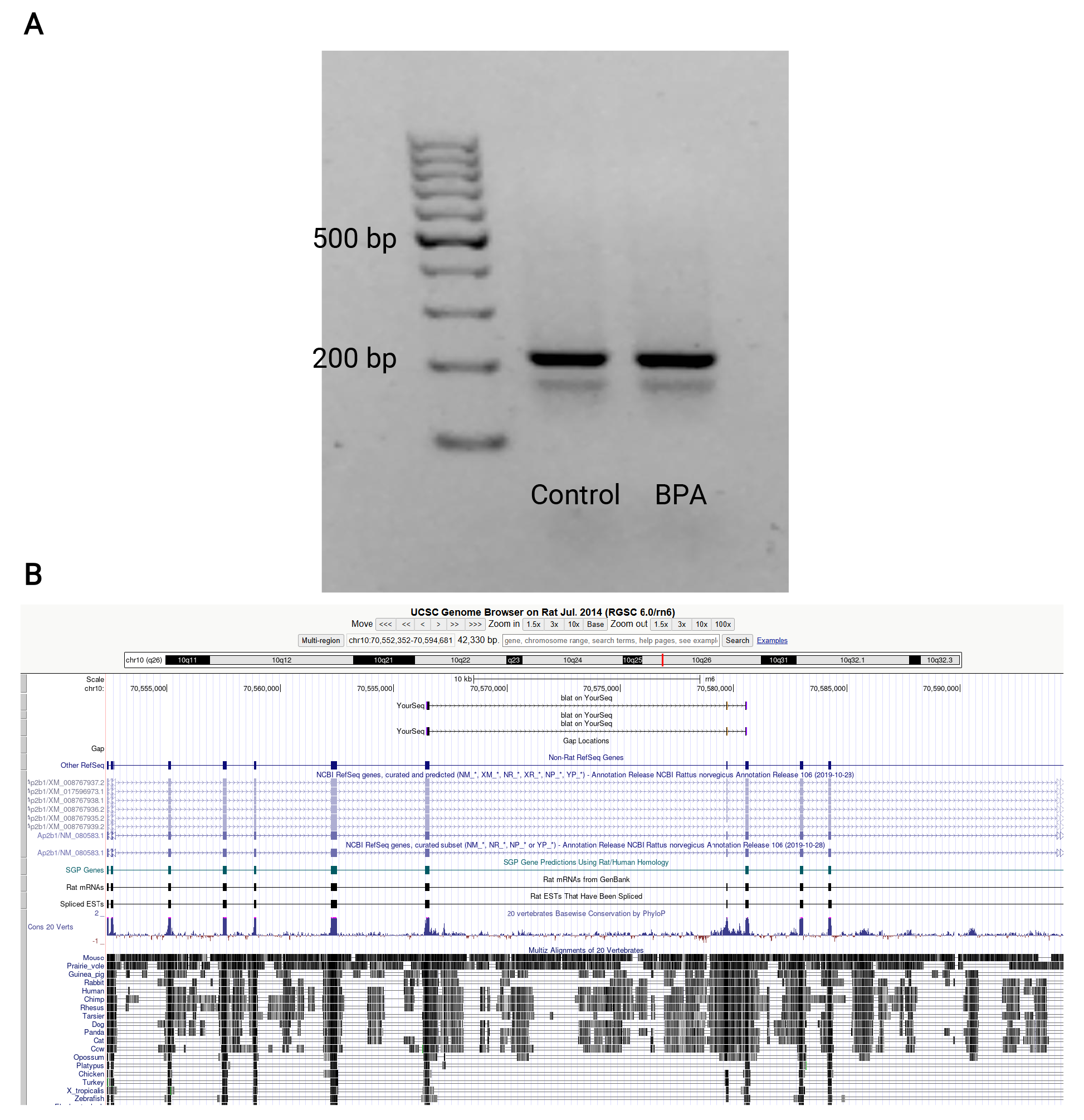
**

**Supplementary Figure 3 Validation of the sequences of the qPCR-HRM products of *Ap2b1***

(A) Gel electrophoresis of *Ap2b1* showing multiple products. The first band from below was 176 bp product, and the second band was 218 bp product., (B) BLAT results of the 218 bp of *Ap2b1* sequencing from Sanger’s sequencing revealed the match sequences of the 218 bp product and the theoretical product from the in-silico PCR test.

**
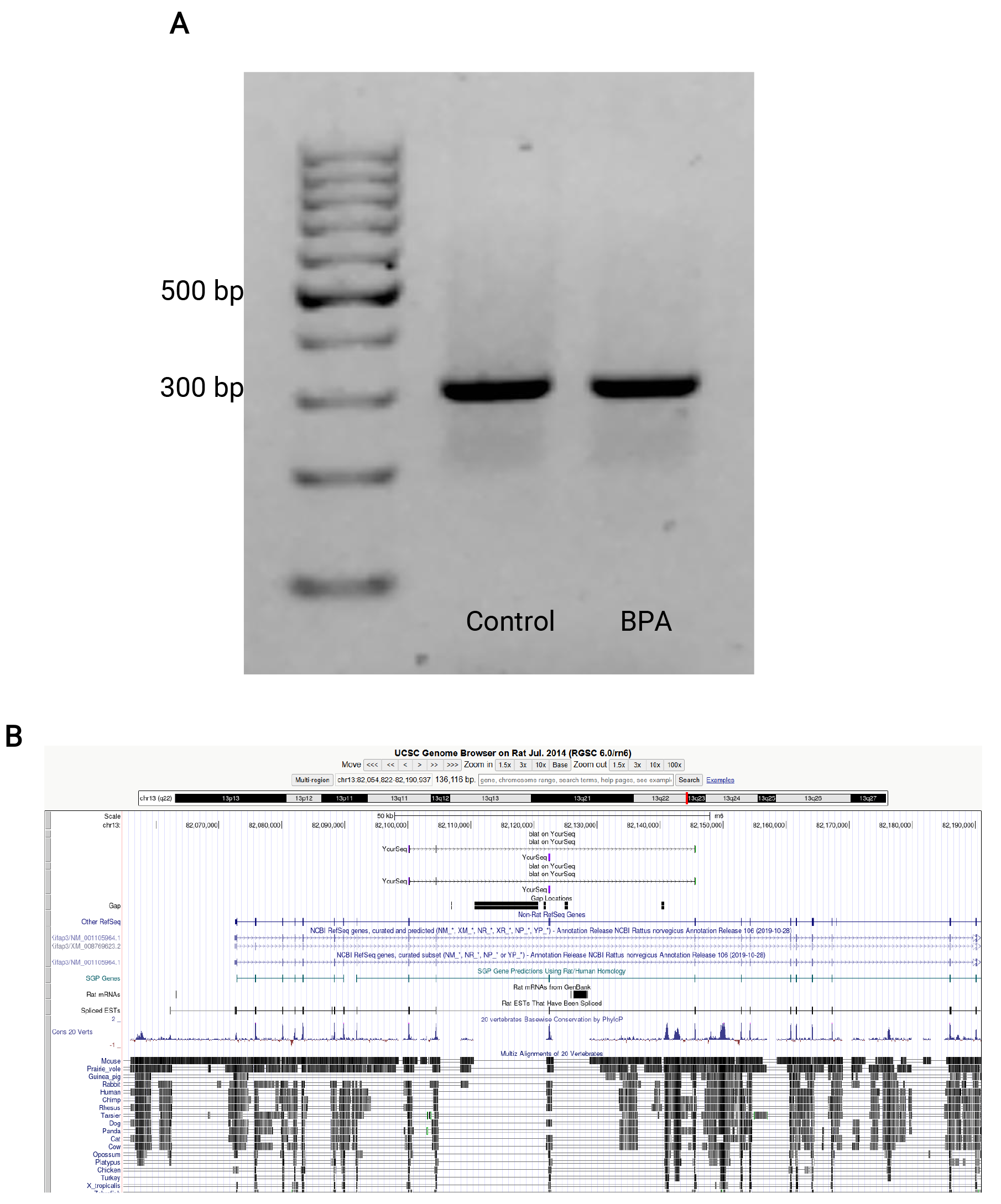
**

**Supplementary Figure 4 Validation of the sequences of the qPCR-HRM products of *Kifap3***

(A) Gel electrophoresis of *Kifap3* showing multiple products (300 bp), (B) BLAT results of the 300 bp of *Kifap3* sequencing from Sanger’s sequencing revealed the match sequences of with the theoretical product from the in-silico PCR test.
